# Development of the Perceived Physical Literacy Questionnaire (PPLQ) for the adult population

**DOI:** 10.1016/j.jesf.2023.09.003

**Published:** 2023-10-05

**Authors:** Peter Holler, Johannes Carl, Mireille N.M. van Poppel, Johannes Jaunig

**Affiliations:** a FH JOANNEUM - University of Applied Sciences, Institute of Health and Tourism Management, Kaiser-Franz-Josef-Straße 24, 8344, Bad Gleichenberg, Austria; b Sport Science Laboratory, FH JOANNEUM - University of Applied Sciences, 8344, Bad Gleichenberg, Austria; c University of Graz, Institute of Human Movement Science, Sport and Health, Mozartgasse 14, 8010, Graz, Austria; d Friedrich-Alexander University Erlangen-Nürnberg, Department of Sport Science and Sport, Gebbertstraße 123b, 91058, Erlangen, Germany

**Keywords:** Physical literacy, Physical activity, Adults, Validation, Questionnaire, Confirmatory factor analysis

## Abstract

**Background/objective:**

In physical literacy (PL) research, instruments for the adult population covering all relevant domians are currently lacking in German language. Therefore, the Perceived Physical Literacy Questionnaire (PPLQ) was developed as an assessment instrument of PL for the adult population. The purpose of this study is to describe the multistage development process leading to the aim to evaluate the psychometric properties of the PPLQ.

**Methods:**

Based on established questionnaires (subscales) operationalizing the six defined PL domains (motivation, confidence, physical competence, knowledge, understanding, and physical activity behavior), we generated a large item pool. Exploratory analyses on survey data (n = 506), compelemented through an expert panel, served to identify the best fitting items. Cognitive interviews (n = 7) and a language certification process (level A2) helped to enhance the content validity of the items. Finally, we assessed the hypothesized factor structure of the PPLQ and its convergent validity with the Physical Activity-related Health Competence (PAHCO) questionnaire in a second independent sample.

**Results:**

Valid data of 417 adults (66% women, 48 ± 16 years) entered the confirmatory factor analysis. We found empirical support for a theory-compatible 24-item version, after reducing complexity (i.e., domain subscales). Additionally, the six domains could be subsumed under an overall factor for PL (χ^2^_247_ = 450.70, χ^2^/df = 1.83, CFI_Robust_ = 0.895, RMSEA_Robust_ = 0.074 [CI_90_ = 0.063–0.085], SRMR = 0.064). Factor loadings, composite reliability, and discriminant validity were sufficient, while acceptable convergent validity was achieved for the total PL score and three domains.

**Conclusion:**

The 24-item version of the PPLQ is appropriate for assessing PL among adults. However, some items (especially in the knowledge domain) can benefit from refinement in further studies.

## 1 Introduction

The concept of physical literacy (PL) has gained worldwide attention over the last two decades^1^^,^^2^ as it is broadly understood as the gateway for sustained lifelong participation in physical activity (PA).^3^^,^^4^ Pioneered by Margaret Whitehead,^5^ the International Physical Literacy Association (IPLA) has defined PL as “the motivation, confidence, physical competence, knowledge and understanding to value and take responsibility for engagement in physical activities for life”.^6^ Noteworthy, the multidimensional concept of PL follows a mind-body integrated, holistic approach to PA and is framed as a lifelong “journey” that every individual takes throughout its life.^2^^,^^7^ Given its ultimate goal of lifelong PA participation,^8^ PL is conceptually linked to a wide array of health outcomes.^9^ Most importantly, by considering several theory-based PA correlates of well-established constructs (e.g., confidence [i.e., self-efficacy] and motivation derived from social cognitive theory or self-determination theory, respectively),^10^, ^11^, ^12^ the PL concept may present an innovative framework to promote PA in different populations and settings.^13^ Particularly in German-speaking countries, such as Austria, where PL has attracted only little attention so far,^14^^,^^15^ the integration of the PL approach could therefore be a promising strategy to reduce the high prevalence of physically inactive adults.^16^ In this context, however, the recognition of its potential role to foster PA among the adult population remains relatively underdeveloped, not just in Austria, but also across the globe.^17^, ^18^, ^19^ To date, the majority of research initiatives have aimed at children, adolescents and recently adults in educational context,^17^^,^^20^, ^21^, ^22^, ^23^ even though PL is in its philosophical essence a concept for all people regardless of age, gender, ethnicity, and socioeconomic status.^24^^,^^25^ This is an important research gap, as it bears the risk of uncoupling PL from its original conception as a lifelong journey.^26^

As the consideration of PL might act as an essential strategy for promoting PA in adults, reliable and valid PL instruments are required for multiple professions and in multiple contexts.^27^ For example, outcomes may enable researchers and practitioners to understand adults' PL levels so that they can implement or evaluate an appropriate intervention.^20^ Furthermore, the results can be used on the policy level to promote and allocate resources to PL initiatives.^27^ Therefore, PL assessment instruments for adults are needed that support the conceptual and theoretical assumptions underlying the PL construct.^9^ In the long term, instruments can help to accumulate empirical findings which are at the moment, to a large degree, only available for children.^17^

At present, there are only a few PL assessment instruments available for adults.^28^ Among these, some have not yet been validated^3^^,^^29^ or do not reflect the holistic nature of the concept by focusing solely on physical aspects.^30^ Only studies on the College Student Physical Literacy Questionnaire (CSPLQ),^31^ the Perceived Physical Literacy Instrument (PPLI),^32^ as well as its adapted versions (e.g., for Simple Chinese,^33^ Spanish,^34^ Turkish^35^ or Persian^36^) have reported satisfactory results for reliability levels and global model fits. However, the target population for both the CSPLQ and PPLI was restricted to specific subgroups of adults within the Chinese culture, namely college students and physical education teachers.^31^^,^^32^ Thereby, all PPLI adaptations relied on the same 18-item pool used to develop the original version (see Table 7 in Mendoza-Muñoz et al.^34^), but extracted different items nested within different factor structures in their final versions, respectively. Moreover, the PPLI and its adaptions have a thematic focus on sport and considered only three domains (factors), which may be a narrow interpretation of PL given the complexity of the concept. In fact, the authors of the Spanish PPLI version,^34^ suggested that further studies should go beyond the three-factor structure of the PPLI by additionally examining other domains in the assessment of PL. For example, the PPLI does not consider PA behavior, even though it is acknowledged as a valuable indicator for the operationalization of PL.^37^ Not only in the PPLI, but also in several other existing PL instruments, two distinct theoretical constructs such as motivation and confidence were combined into one domain within the operationalized factor structure (see e.g.,^32^^,^^34^^,^^37^). This combination may limit both the interpretation of the domain score^38^ and the implementation of an appropriate PL-based intervention according to the assessment results.^20^ For instance, a limited level of motivation requires similar but still different intervention strategies than a limited level of confidence (i.e. self-efficacy) and vice versa.^39^^,^^40^ It is to be noted that no existing instrument for adults provides an overall PL score, which allows quantification of an individual's “PL level” – a term referenced very frequently in literature.

The current state of published literature led us to conclude that to date there is no PL assessment instrument for adults available, which is adaptable into the German language and the cultural setting. Most notably, no existing instrument allows for a comprehensive and differentiated measurement of all key attributes according to the IPLA definition (motivation confidence, physical competence, knowledge, understanding and PA behavior) and integrates a composite score for PL. Hence, our overall aim was to develop the Perceived Physical Literacy Questionaire (PPLQ); a PL assessment instrument for the general adult population (i.e., aged 18–65 years with diverse socioeconomic and PA background), which covers all these six domains separately and additionally subsume them under an overall PL factor. In this sense, the instrument should be easy-to-understand (i.e., at least A2 language level),^41^ and easy-to-administer as well as cost- and time-efficient in terms of applications within large-scale assessments. The specific aims in this multistage process were to (i) assemble a comprehensive item pool, with items suitable for assessment among the adult population and (ii) to identify the most appropriate items based on data of an online survey. Subsequently, we aimed to (iii) evaluate the content validity of the remaining items with cognitive interviews in a target group and (iv) by external comprehensibility inspection. In the last stage (v), we evaluated how well the shortened version fitted a hypothesized measurement model in a second, independent sample and undertook further improvements, if indicated.

## 2 Methods

### 2.1 Development of the PPLQ

The development of the PPLQ was conducted in Austria, in German language. Its development process started in 2019 and included five stages, whereas the main text of this paper focuses on stage 5. The aims, methods and results of the preceding stages are also summarized below. A detailed description for each stage can be found in Appendix A.

#### 2.1.1 Stage 1

The development of the PPLQ is based on a questionnaire used in previous PL-intervention trials.^42^^,^^43^ This instrument already consisted, to a large extent, of a conglomerate of established questionnaires (subscales) for the respective PL domains. Yet, their psychometric properties have not been investigated for application in an integrative model and multidimensional measurement framework. Accordingly, this is crucial to evaluate whether the underlying constructs for the domains justify separate treatment and are distinct from each other (i.e., sufficient discriminant validity). Moreover, we identified methodological and content-related shortcomings during the trials at the domain and global level of the instrument (e.g., reliability of the domain knowledge; scoring procedure; no measurement of physical competence, which is, however, an important part of current PL definitions^6^). In autumn 2019 we revised this initial questionnaire according to our proposed six-domain PL measurement model (see above). Since the development of a questionnaire based on a multidimensional model is always a challenge,^44^^,^^45^ the aim of stage 1 was to generate a large item pool for our underlying PL domains. Within this procedure, we retained questionnaires (subscales) from our previous instrument, if considered appropriate and used newly validated questionnaires (subscales) based on a literature review. We could not identify any established questionnaires (subscales) for understanding and knowledge fitting the PL framework and the subscales in our former questionnaire were found to be inappropriate (i.e., some items in the understanding domain were thematically focused on motivation and confidence, and the knowledge domain was operationalized by open-ended questions).^42^^,^^43^ Therefore, we additionally adjusted item wordings and self-constructed new items for these domains. In total, the questionnaire version from stage 1 (referred as PPLQ version 1) consisted of 61 items (see Appendix A, pages 2 to 4).

#### 2.1.2 Stage 2

The aim of this stage was to identify the most convenient items from the generated item pool in stage 1. For this purpose, we conducted a cross-sectional study in winter 2019, in which a sample of 506 students and university staff members (72% female, 27.31 ± 10.11 years), who did not participate in any of the following development stages completed the PPLQ version 1. The subsequent item selection procedure followed a two-step-approach, each under the premise to have a maximum of two subscales per domain with a minimum of three items within a subscale. In a first step, we conducted exploratory factor and reliability analyses for each latent domain, except for PA behavior as it is not operationalized by a reflective measurement model. The subscales/items with the most appropriate measurement properties (i.e., factor loadings and Cronbach's alpha coefficients) were selected.^46^ In a second step we critically discussed the results with seven experts from research and practice (three senior and four early-stage researchers all working in the field of PA and/or public health and familiar with the PL concept), to select the items/subscales with the highest content validity. If applicable, we used the content validity index (CVI) as a well-established method to calculate content validity quantitatively.^47^ Applying the CVI, the experts rated the subscales/items of one domain either as “relevant (1)” or “non relevant (0)”, whereby a CVI of at least 0.83 (i.e., at least six out of seven experts gave a rating as “relevant”) was considered as satisfactory.^48^ In total, this procedure resulted in a PPLQ version 2 of 34 items. Appendix A (pages 5 to 13) provides a detailed description of the methods and results of stage 2.

#### 2.1.3 Stage 3

Within this stage, we inspected the remaining items from the PPLQ version 2 using cognitive interviewing, which is a method for assessing the content clarity and interpretation consistency of self-reported items based on respondents' thought processes while answering the items.^49^ Adapting a theoretical sampling strategy,^50^ two rounds of a total of seven cognitive interviews (n_1_ = 3, n_2_ = 4) were conducted (71% female, 54.71 ± 2.29 years). The analysis of the interviews from round two indicated a saturation (i.e., no new information was provided). All results were discussed involving the expert committee from stage 2 to achieve a consensus on which changes should be made (see Appendix A, pages 13 to 15).

#### 2.1.4 Stage 4

Subsequently, the PPLQ version 3 was subjected to an external comprehensibility check by *capito* (www.capito.eu), a certified organization for barrier-free and comprehensible information and communication. As part of this process, the PPLQ was modified (see Appendix A, Table A.12) and certified to A2 language level, which allows the PPLQ to be classified as an easy-to-understand questionnaire (i.e., 96% of the Austrian population should be capable of understanding it).^41^ Overall, the modifications conducted within the four stages resulted in a PPLQ version 4 with 31 items and six domains (see Appendix A, pages 16 to 21). Table 1 shows the item labels, the English item wordings, and assigned response scales for each item of the PPLQ version 4. The full PPLQ version 4 in the original German version can be found in Appendix B.

**Table 1:** Item labels and wordings of the PPLQ version 4 (i.e., 31 item version).

**Domain:** Physical competence [PCO] **Two subfactors:** strength [PCO-ST] & endurance [PCO-EN] **Response scale:** 6-point Likert scale (strongly agree - strongly disagree)	
**Label**	**Items (in order of appearance)**
PCO_ST1	I have a lot of muscle power.
PCO_EN1	I can run for at least 30 min minutes without stopping.
PCO_ST2	It is easy for me to lift heavy objects (e.g., full beverage crate).
PCO_ST3*	I would do well in a test of muscle strength.
PCO_EN2*	I can be physically active for a long period of time without getting tired.
PCO_EN3	I am good at endurance activities (e.g., distance running, aerobics, cycling, swimming, or cross-country skiing).
**Domain:** Understanding [UND] **Response scale:** 6-point Likert scale (strongly agree - strongly disagree)	
**Label**	**Items (in order of appearance)**
UND1	I see a purpose in engaging in physical activity regularly.
UND2	I feel a lot of appreciation for people engaging in regular physical activity.
UND3	I think initiatives in companies to increase physical activity (e.g., company walking day) make sense.
**Domain:** Motivation [MOT] **Two subfactors:** intrinsic motivation [MOT-IN] & identified motivation [MOT-ID] **Response scale:** 6-point Likert scale (strongly agree - strongly disagree) **Introductory sentence:** “I plan to be physically active on a regular basis in the coming weeks and months …”	
**Label**	**Items (in order of appearance)**
MOT_IN1	… because I simply enjoy it.
MOT_ID1	… because the positive consequences are simply worth the effort.
MOT_IN2*	… because physical activity is simply part of my life.
MOT_ID2*	… because it is good for me.
MOT_IN3	… because it gives me experiences that I wouldn't want to miss.
MOT_ID3	… because I have good reasons for it.
**Domain:** Confidence (i.e., self-efficacy) [CON] **Two subfactors:** internal barriers [CON-IB] & external barriers [CON-EB] **Response scale:** 6-point Likert scale (strongly agree - strongly disagree) **Introductory sentence:** “I still engage in planned physical activities even if …”	
**Label**	**Items (in order of appearance)**
CON_IB1*	… I am tired.
CON_IB2	… I feel depressed.
CON_IB3*	… I am annoyed about something.
CON_EB1	… I can't find anyone to do sports with me.
CON_EB2	… weather is bad.
CON_EB3	… an interesting TV program is running.
**Domain:** Physical Activity Behavior [PAB] **Response scale:** open response categories (unit for part a of each PAB-item - “days per week”, unit for part b of each PAB-item “minutes per day”). At each PAB-item there was also an addition response category “no walking”, “no vigorous-intensity activity” and “no moderate-intensity activity “, respectively.	
**Label**	**Items (in order of appearance)**
PAB1	a. During the last 7 days, on how many days did you walk for at least 10 min minutes at a time? This includes walking distances at home or at work, walking to get from one place to another, and all other walking for recreation, exercise, or leisure. b. How much time did you usually spend walking on one of those days?
PAB2	a. Think only about those physical activities that you did for at least 10 min minutes at a time. During the last 7 days, on how many days did you do vigorous physical activities like aerobics, running, fast cycling or fast swimming? b. How much time did you usually spend doing vigorous physical activities on one of those days?
PAB3	a. Think again only about those physical activities that you did for at least 10 min minutes at a time. During the last 7 days, on how many days did you do moderate physical activities like carrying light loads, bicycling at a regular pace, or swimming at ordinary speed? Caution: This does not include walking! b. How much time did you usually spend doing moderate physical activities on one of those days?
Table 1 **(continued):** 31-items version of the PPLQ from stage 4 (i.e., PPLQ version 4)	
**Domain:** Knowledge [KNO] **Two subfactors:** how to move [KNO_HM] & knowledge of the benefits [KNO_KB] **Response scale:** closed response categories (since the scales are different for each item, they are reported below together with the item description - italics, correct answer in bold)	
**Label**	**Items (in order of appearance)**
KNO_HM1	Up to what age is muscle strength trainable? *[40, 50, 60, 70, 80, 90,****always****]*
KNO_HM2*	According to the Austrian physical activity guidelines, at least how many minutes per week should you perform activities that involve a slight increase in breathing and pulse rate e.g., brisk walking)? *[*30* *min *(½ hour),* 45* *min *(¾ hours),* 60* *min *(1 h hour),* 75* *min *(1 ¼ hours),* 90* *min *(1½ hours),* 120* *min *(2 h hours),***150* *min *(2½ hours)****,* 180* *min *(3 h hours),* 240* *min *(4 h hours)]*
KNO_HM3	Pure strength training (without endurance training) also has health benefits. *[****true****, false]*
KNO_KB1	A physically inactive lifestyle increases the risk of suffering the following diseases: *[****breast cancer, dementia, hypertension****] – multiple choice response scale*^*a*^
KNO_HM4*	Women need different strength exercises than men to build muscle. *[true,****false****]*
KNO_KB2	Physical activity can improve the course of the following diseases: *[****sugar disease (diabetes mellitus type II), Parkinson's disease, joint wear (arthrosis), heart failure****] - multiple choice response scale*^*b*^
KNO_HM5*	Strength training is suitable for losing weight (body fat). *[****true****, false]*

***Note:*** * item excluded in the final 24-item version from stage 5 (i.e., PPLQ version 5).

***Note:*** * item excluded in the final 24-item version from stage 5 (i.e., PPLQ version 5); ^a^ in the analysis procedure each response category is counted as one single item with “breast cancer” = KNO_KB1a, “dementia” = KNO_KB1b and “hypertension” = KNO_KB1c [excluded in the final 24-items version], ^b^ in the analysis procedure each response category is counted as one single item with “sugar disease” = KNO_KB2a, “Parkinson's disease” = KNO_KB2b, “joint wear” = KNO_KB2c and “heart failure” = KNO_KB1d.

#### 2.1.5 Stage 5

The focus of the current study relates to this stage, in which the aim was to evaluate the hypothesized factor structure and psychometric properties of the PPLQ version 4 with an independent sample (see next paragraph). We tested the theoretically assumed factor structure with confirmatory factor analyses (CFAs), initially for the entire model and subsequently for each domain separately. In cases of misfits, the model was modified based on content-related and statistical considerations. Moreover, we examined the convergent validity of the PPLQ by employing the Physical Activity-related Health Competence (PAHCO)^51^^,^^52^ questionnaire. The corresponding procedure is described in more detail in the result section.

### 2.2 Design, participants, and procedures

The following descriptions exclusively refer to stage 5 as the empirical core process of the entire PPLQ development. Within this stage, a cross-sectional study was conducted, including adults who were (i) aged between 18 and 65 years and (ii) fluent in the German language. We collected date between February 2021 and April 2022. Eligible participants completed the paper-pencil version of the PPLQ version 4, with a subsample also completing the PAHCO^51^^,^^52^ questionnaire to assess convergent validity. In addition, we gathered information on gender, age, and education level. Participants were recruited from two settings: (i) via four primary health care centers in Styria, a province of Austria (participants of this subsample completed both the PPLQ and PAHCO), (ii) via personal contact during “physical activity education events”, which were held in six rural Styrian communities as part of the project “MOVEluencer”.^53^ This subsample only completed the PPLQ, since the time frame of the events did not allow to complete both the PPLQ and PAHCO. The study was approved by the Research Ethics Committee of the University of Graz, Styria, Austria (GZ.39/101/63 ex 2019/20) and informed consent was obtained from all participants.

### 2.3 Scoring of the PPLQ

Domain scores for each of the six domains and a total PL score are calculated as follows. The domain scores range between 0 and 100, with higher values representing a greater domain proficiency. Acknowledging the holistic nature of the PL concept, the total PL score represented a composite calculation in which each of the six domains were weighted equally with 16.67%. This composite score also ranged between 0 and 100, with higher values indicating a better PL. Note that the domain score for PA behavior is derived from a theory-based nonlinear saturation function, with 150 min of moderate-to-vigorous PA (MVPA) per week corresponding to a score value of 66 and 300 min of MVPA per week corresponding to a value of 100, respectively.^54^, ^55^, ^56^ A detailed description of the scoring procedure can be found in Appendix C, both for the PPLQ version 4 (Table C.1) and for the final version resulting from stage 5 (PPLQ version 5; Table C.2).

### 2.4 Convergent validity

We employed the PAHCO^51^^,^^52^ questionnaire to assess the convergent validity of the PPLQ. Conceptualized as a multidimensional framework at the intersection of PL and health literacy, the underlying PAHCO model is theoretically and conceptually closely related to the PL approach.^57^ More specifically, the PAHCO model assumes that three interrelated sub-competencies are required for a healthy, physically active lifestyle: (i) movement competence, (ii) control competence, and (iii) self-regulation competence. Within the PAHCO questionnaire these three competences are posited as second-order factors bundling 10 first-order factors with a total of 42 items.^51^^,^^52^ In collaboration with a member of the PAHCO development team (JC), we expected equivalent scores of the PAHCO to be highly correlated with the PPLQ (ρ ≥ 0.5) in case of sufficient evidence for convergent validity. With regard to the PAHCO, we only used the total score of the 3 second-order factors for determining convergent validity. For instance, we expected high correlations between the PAHCO scores from the second-order factor “movement-competence” with the domain score “physical competence” of the PPLQ. The theoretically assumed correlations, as well as the whole intercorrelation matrix (for exploratory purposes) is presented in the corresponding result section.

### 2.5 Statistical analysis

The collected data was accurately screened by excluding participants with implausible and missing values. More specifically, we excluded only participants with missing values for all items in one or more domains (PPLQ) or first-order factors (PAHCO). The remaining missing values were imputed using multiple imputation, whereby the result of five imputed data sets were averaged to have one single complete data set.^58^ We applied CFAs for factorial validity assessment. The domain PA behavior entered the CFA with a single indicator given the formative nature of this measurement model^59^ and the categorical character of the variable with eleven levels (linear transformation from the MVPA score). The multiple-choice items of the knowledge domain were partitioned into separate dichotomous items (true/false). We used the mean and variance adjusted weighted least squares (WLSMV) estimator, which is recommended for models with binary and categorical data. For the assessment of the global model fit, we relied on the following indices: comparative fit index (CFI), root mean square error of approximation (RMSEA) and standardized root mean square residual (SRMR).^60^ Since fit indices^60^ under the WLSMV estimator have a profound tendency to mask model misspecification,^61^ we relied on the robust versions of CFI and RMSEA proposed by Savalei.^62^ Furthermore, we adopted a variant which considers the underlying degrees of freedom (χ^2^/df) as the chi-square (χ^2^) test is highly sensitive to model complexity and sample size.^62^ We applied following rules of thumb for first indication of adequate model fit. A good model fit was considered when χ^2^/df ≤ 2.0, CFI_Robust_ ≥ 0.95, RMSEA_Robust_ ≤ 0.05 and SRMR ≤ 0.05 and an acceptable model fit when χ^2^/df ≤ 3.0, CFI_Robust_ ≥ 0.90, RMSEA_Robust_ ≤ 0.08 and SRMR ≤ 0.10.^63^ Factor loadings of ≥ 0.5 for newly developed items (knowledge and understanding), and of ≥ 0.6 for established items (motivation, confidence and competence) were treated as adequate.^64^^,^^65^ We inspected the models in more detail on the local level using modification indices, residual correlations, and item difficulties. Composite reliability was assessed by means of McDonald's omega (ω), with values exceeding 0.7 indicating an acceptable level of internal consistency.^66^ We derived evidence of discriminant validity based on the Fornell-Larcker criterion.^67^ Accordingly, discriminant validity is established if the average variance extracted (AVE) by a construct (i.e., latent variable) is greater than the squared correlation between the construct and any other constructs. For the convergent validity assessment between the PPLQ and the PAHCO, spearman correlation coefficients (ρ) were applied. The analyses regarding the CFAs and the reliability metrics were conducted in R (4.2.2)^68^ via the packages lavaan (version 0.6–13)^69^ and semTools (version 0.5–6).^70^ For all other statistical analyses, we used IBM SPSS 27.

## 3 Results

### 3.1 Participants

A total of 476 participants completed the PPLQ version 4, with a subsample of 244 participants also completed the PAHCO. Regarding the PPLQ, 43 participants were excluded as they had missing values for all items in one or more domains. Another 16 participants were excluded as they reported implausible (contradictory) data within the PA behavior domain (e.g., 0 days and 5 h of moderate PA per week). Hence, the final sample for the factorial validity assessment of the PPLQ consisted of 417 participants. The percentage of imputed missing values for the PPLQ was 0.26% (39 values).

Participants excluded from the PPLQ were also excluded from all analyses related to the PAHCO (n = 40). Additionally, we excluded 11 participants as they had missing values for all items in one or more first-order factors and another 35 participants because of implausible (contradictory) data. Hence, for the convergent validity assessment (PPLQ vs. PAHCO) the data of 158 participants was used. The percentage of imputed missing values for PAHCO was 0.89% (74 values). Participants characteristics for the total sample and the subsample completing also the PAHCO are illustrated in Table 2, with group differences only observed for education level.

**Table 2:** Participants characteristics.

Characteristics	Total sample (n = 417)	PAHCO-subsample (n = 158)	*p-*value
**Gender (female)**^**a**^	274 (66%)	98 (62%)	0.441
**Age (years)**^**b**^	48.10 ± 16.58	46.31 ± 15.90	0.261
**Education Level n (%)**^**c**^			
Compulsory school	28 (7%)	5 (3%)	0.042*
Apprenticeship	108 (26%)	42 (27%)	
Vocational middle school	67 (16%)	21 (13%)	
High school (A-Level)	77 (18%)	25 (16%)	
University-related teaching institution	33 (8%)	7 (4%)	
University	102 (25%)	58 (37%)	

***Note:***^a^ missing data for one participant, both in the total sample and in the PAHCO-subsample; ^b^ missng data for 23 participants in the total sample and 19 in the PAHCO-subsample; ^c^ missing data for two participants in the total sample and no missing data in the PAHCO-subsample. * significantly different at the α = 0.05 level

### 3.2 Factorial validity

First, we tested our assumed 31-item model of the PPLQ version 4. As visualized in Figure A.1 (Appendix A), this model was specified as a hierarchical model with a third order g-factor for overall PL. Regarding the IPLA definition,^6^ this structure may provide the most interpretable solution as well as the closest conceptual representation of PL. Using the WLSMV estimator, however, the CFA could not be computed. Subsequently, further CFAs were conducted separately for the respective PL domains. Heywood cases were observed related to potential autocorrelations between indicators (i.e., observed variables) in the model.^71^ Therefore, the model was modified based on content and statistical considerations (i.e., fit indices, factor loadings, modification indices and item difficulties). Redundant and inadequate items were removed, and sub-domains merged into one factor per domain. The final modified models on domain level revealed acceptable model fits (see Appendix D, Table D.1). This procedure resulted in an overall 24-item second-order model with PL as a g-factor (referred as PPLQ version 5). The global model-fit of the final model was acceptable (χ^2^ 247 = 450.70, p < 0.001, χ^2^/df = 1.83, CFI = 0.974, CFI_Robust_ = 0.895, RMSEA = 0.045 [CI_90_ = 0.038–0.051], RMSEA_Robust_ = 0.074 [CI_90_ = 0.063–0.085], SRMR = 0.064]. Even so, there were still some minor statistical discrepancies with six modification indices greater than 20 and residual correlations greater than 0.2 between the item PCO_ST1 and PAB_MVPA, KNO_KB2a and KNO_HM3 as well as KNO_KB2a and KNO_KB1a. Notably, the correlated residuals were not permitted in the final model.

**Fig. 1:**
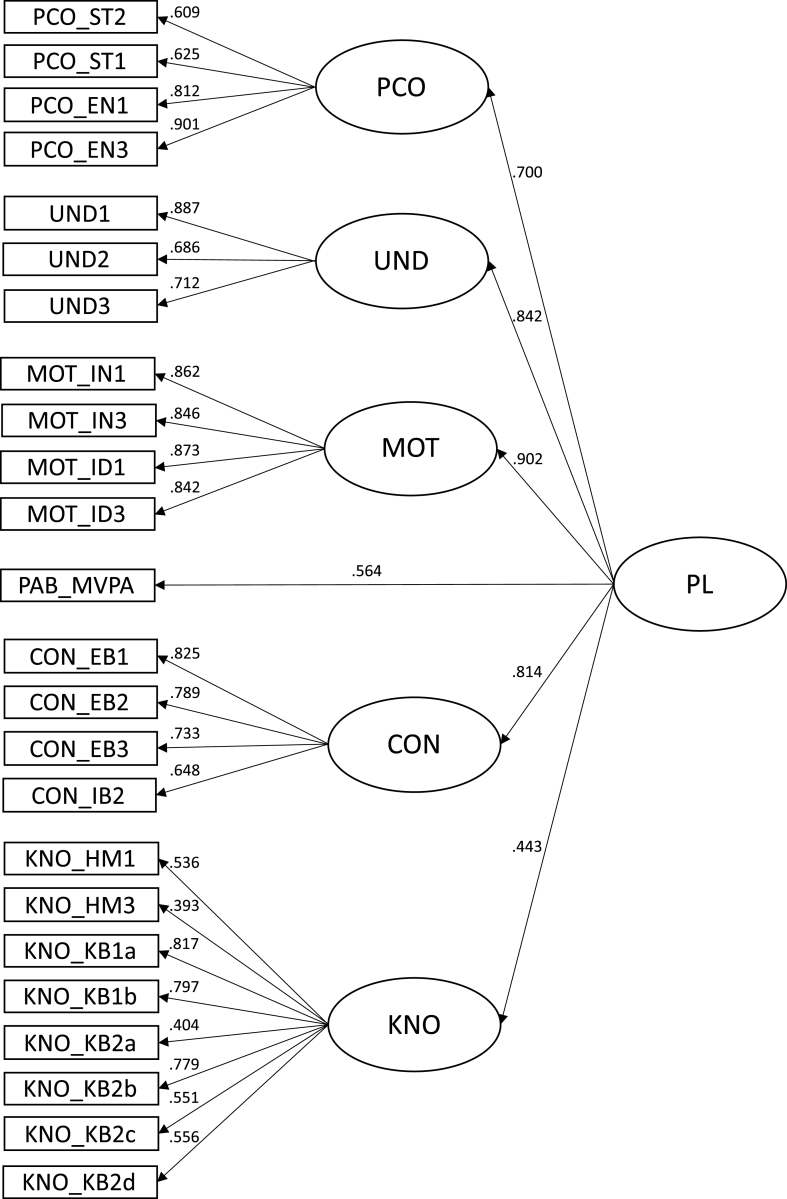
24-item second-order PPLQ model with PL as a g-factor. PL: physical Literacy; PCO: domain physical competence; UND: domain understanding; MOT: domain motivation; CON: domain confidence (i.e., self-efficacy); KNO: domain knowledge; the full wording of the corresponding item-labels can be retrieved from Table 1.

For the final 24-item-model, all factor loadings were found to be highly significant (p < 0.001) and acceptable, excepted for KNO_HM3 (0.393) and KNO_KB2a (0.404; see Fig. 1). Considering the internal consistency, all coefficients for the domains (subscales) ranged from sufficient to good (0.70 ≤ ω ≤ 0.90; see Table 3). Additionally, the omega coefficient for the g-factor (i.e., overall PL) was very satisfactory (ω = 0.90). Except for the domain knowledge (AVE = 0.39), the AVEs for all other domains were in an acceptable range (0.56 ≤ AVE ≤ 0.73). Moreover, the AVE values of all domains exceeded the corresponding highest squared correlation estimates, indicating sufficient discrimination between the domains. Descriptive statistics for the final 24-item-version of the PPLQ are presented in Table 4, Table 5, showing mean, standard deviation, minimum, maximum, skewness, kurtosis and item difficulty in terms of the knowledge domain, respectively.

**Table 3:** Analyses of reliability and discriminant validity.

Domains	Composite reliability (ω)	AVE	Highest squared correlation
**Physical competence**	0.818	0.557	0.399
**Understanding**	0.728	0.588	0.577
**Motivation**	0.882	0.733	0.577
**Confidence(i.e., self-efficacy)**	0.817	0.565	0.539
**Physical Activity Behavior**	n.a.	n.a.	n.a.
**Knowledge**	0.695	0.391	0.160

**Note:** ω: McDonald's omega; AVE: average variance extracted. The domain physical activity behavior was not included in the analyses as it is represented only by one indicator (see Fig. 1 and section 2.5 in the manuscript, respectively).

**Table 4:** Descriptive statistics of the PPLQ version 5 (i.e., 24-item version; except domain knowledge).

Domain	Item	*M*	*SD*	*Min*	*Max*	*Skew*	*Kurt*
**Physical competence**	PCO_ST_1	3.04	1.09	0	5	−0.49	0.36
	PCO_EN_1	2.47	1.77	0	5	0.04	−1.35
	PCO_ST_2	3.10	1.54	0	5	−0.59	−0.70
	PCO_EN_3	2.62	1.61	0	5	−0.23	−1.05

**Understanding**	UND_1	4.47	0.83	0	5	−2.03	5.11
	UND_2	4.34	0.87	0	5	−1.65	3.71
	UND_3	4.13	1.12	0	5	−1.66	2.90

**Motivation**	MOT_IN_1	3.93	1.08	0	5	−0.99	0.82
	MOT_ID_1	4.18	0.88	0	5	−1.21	2.24
	MOT_IN_3	3.76	1.14	0	5	−0.99	0.84
	MOT_ID_3	4.07	0.99	0	5	−1.47	2.93

**Confidence (i.e., self-efficacy)**	CON_IB_2	2.81	1.34	0	5	−0.35	−0.60
	CON_EB_1	3.26	1.50	0	5	−0.55	−0.66
	CON_EB_2	2.92	1.41	0	5	−0.30	−0.74
	CON_EB_3	3.02	1.61	0	5	−0.34	−1.03

**Physical Activity Behavior**	Total_MVPA	281.38	426.31	0	4320	4.13	25.98
	Total_MVPA_Score_*	58.71	38.71	0	100	−0.31	−1.46

***Note:*** * variable ranging between 0 and 100 (see section 2.3 in the manuscript for detailed information); M: Mean; SD: Standard deviation; Min: Minimum; Max: Maximum; Skew: Skewness; Kurt: Kurtosis. The full wording of the corresponding item-labels can be retrieved from Table 1.

**Table 5:** Descriptive statistics of the PPLQ version 5 (i.e., 24-item version) - domain knowledge.

Domain	Item	Item difficulty (% correct answered)
**Knowledge**	KNO_HM1	79.6
	KNO_HM3	81.1
	KNO_KB1a	20.6
	KNO_KB1b	54.2
	KNO_KB2a	81.5
	KNO_KB2b	41.2
	KNO_KB2c	70.5
	KNO_KB2d	80.1

***Note:*** The full wording of the corresponding item-labels can be retrieved from Table 1.

### 3.3 Convergent validity

The PL total score and the domain scores for physical competence, motivation, and confidence showed sufficient correlations with the corresponding second-order factors of PAHCO. Yet, the correlation coefficients for the domains understanding, PA behavior and knowledge did not reach an acceptable level of convergent validity (see Table 6 for detailed correlations; the whole intercorrelation matrix including also the first-order-factors from the PAHCO can be found in Appendix E, Table E.1).

**Table 6:** Theoretically assumed (spearman's rank) correlations of PPLQ version 5 (i.e., 24-item version) and PAHCO.

	PCO	UND	MOT	CON	PAB	KNO	Overall PL
**PAHCO: second-order-factors**							
movement competence	0.854				0.437		0.719
control competence					0.313	0.279	0.591
self-regulation competence		0.457	0.692	0.591	0.450	0.277	0.701

***Note:*** n = 158 and p < 0.001 for all correlations. Non-expected correlations, including those which were significant, were masked to avoid overinterpretations of convergent validity. The whole intercorrelation matrix can be found in Appendix E, Table E.1. PCO: physical competence; UND: understanding; MOT: motivation; CON: confidence (i.e., self-efficacy); PAB: physical activity behavior; KNO: knowledge; PL: physical literacy; PPLQ: Perceived Physical Literacy Questionnaire; PAHCO: Physical Activity-related Health Competence (questionnaire).

**Table 7:** Description of the meaning of each domain of PPLQ version 5 (i.e., 24-item version).

Domain	Meaning
**Physical competence**	Refers to a person's perception of his/her own fitness and ability to perform various strength and endurance related physical activities.
**Understanding**	Refers to a person's grasp of the value of physical activity for lifelong health and well-being.
**Motivation**	Refers to a person's inherent satisfaction and pleasure to engage in regular physical activity.
**Confidence**	Refers to a person's situational belief in his/her capabilities to adopt and maintain a physically active lifestyle.
**Knowledge**	Refers to a person's knowledge of health-enhancing physical activities and how to perform them. In addition, this refers to a person's knowledge of the health benefits of being physically active.
**Physical activity behavior**	Refers to the extent in which a person performs moderate to vigorous physical activity of all types.

## 4 Discussion

The purpose of this paper was to describe the development process and first validity insights for the PPLQ as an instrument to measure PL in the German-speaking adulthood population. From an initial large item pool for the underlying PL domains, the most convenient items were selected based on exploratory results of a cross-sectional survey and expert ratings. Subsequently, content validity was enhanced by conducting cognitive interviews and an external comprehensibility check. In stage 5 of the study, the hypothesized factor of the PPLQ and its convergent validity with the PAHCO questionnaire^51^^,^^52^ was assessed. We found empirical support for a theory-compatible 24-item version covering all six domains of the IPLA definition,^6^ while not releasing residual correlations as well as applying robust estimators and goodness-of-fit indices.^62^ The six domains could be subsumed under an overall factor for PL, with the domains being distinct from each other (i.e., sufficient discriminant validity) and therefore allowing for independent meaningful interpretation. Evidence of adequate internal consistency was established at the domain level and for the overall factor. An acceptable level of convergent validity was achieved for the total PL score and the domains physical competence, motivation, and confidence but not for understanding, PA behavior and knowledge.

To the best of our knowledge, the PPLQ is the first PL assessment instrument for adults that provides a comprehensive and differentiated measurement of all key attributes of the IPLA definition.^6^ Specifically, we operationalized each theoretical PL attribute separately, leading to the identification of six domains: (i) motivation, (ii) confidence, (iii) physical competence, (iv) knowledge, (v) understanding and (vi) PAbehavior (a description of the meaning of each domain can be found in Table 7). Each domain was equally weighted. From this perspective, the PPLQ may occupy a middle position on a continuum between an idealistic and a pragmatic approach to assess PL.^45^^,^^72^

By contrast, several adaptions of the PPLI used different items in varying factor structures (see Table 7 in Mendoza-Muñoz et al.^34^). For example, in the Spanish adaption,^34^ motivation and confidence have been merged into a single domain. Similarly, in the simplified Chinese adaption,^33^ physical competence and confidence have been grouped together. In summary, such procedures entails the risk that a domain may not be recognized as unique and therefore, may not be interpreted clearly and consistently.^38^ In younger age groups (adolescents) the authors of the Portuguese PL Assessment (PPLA) argued for a combination of scales under the psychological factor but differentiated operationalization and interpretation (e.g., motivation and confidence).^73^^,^^74^ In the adult population, apart from the PPLQ, only the CSPLQ^31^ seems to satisfy the meaningful separation of domains. However, the CSPLQ was developed for Chinese college students, while the entire development process of the PPLQ was focused on the general adult population (i.e., aged 18–65 years with diverse socioeconomic and PA background) within the German-speaking culture. Overall, the PPLQ may provide a valuable approach to ‘translate’ the PL domains into measurable and clearly interpretable latent constructs. In this respect, we encourage other researchers to further develop and adapt the PPLQ (i.e., in other languages).

Based on the results of our study, we endorse an application of the PPLQ in research and practice. However, caution is warranted since some domains may need a refinement in future studies. Regarding the knowledge domain, we observed an insufficient AVE of less than 0.5, while two items had factor loadings below the critical value of 0.5.^64^^,^^65^ The preceding exploratory analyses (from stage 2) also revealed insufficient properties for this domain, which is partially compensated by the higher number of items. When interpreting these findings, it is necessary to consider that knowledge in the context of PL is (and was) constructed as a broad concept.^75^^,^^76^ This procedure may have yielded in a set of items with comparatively moderate factor loadings.^77^ At this point, further studies may conclude that the knowledge domain should rather be operationalized via a formative than a reflective measurement model. In reflective models, the latent construct influences the manifest indicators (i.e., items), which share a common theme and are desirably highly correlated. In formative models in contrast, indicators uniquely define the latent construct, with each contributing to a distinct aspect of the construct and are not necessarily correlated.^78^ In fact, assessing PA knowledge per se is an understudied area across all age groups,^21^^,^^79^^,^^80^ with no validated scale currently available for adults.^77^ Accordingly, there is no clear consensus on the content of the knowledge domain required to implement a formative measurement approach. In summary, further studies are necessary addressing these issues in more detail.

Moreover, further studies are needed to build on the discussion whether PA behavior is an outcome or a domain of PL. For the development of PPLQ we adopted the CAPL developers' interpretation of the IPLA definition: “When people value and take responsibility for engaging in physical activity, they will demonstrate this by being physically active”.^37^ In this context, PA behavior is envisioned as a domain of the PL framework. However, this view seems to contradict the current operational approaches to PL in adults. Longitudinal studies on the causal relationship are needed to undermine the assumption.

The present study has several limitations that should be considered. In our convenience sample, women, middle-aged adults, and higher educated individuals were overrepresented in comparison to the general population. Besides, nearly one third of the study participants (32%) reported over 300 min of MVPA. In this context, we identify the possibility of a selection bias in our recruitment strategy that has disproportionately attracted participants with a special interest in PA and exercise. This might have also affected the results of other domains, especially those with an affective characteristic (e.g., motivation). Consequently, the external validity of our results should be interpreted with caution and further studies with a large representative sample of adults are necessary to generalize the study findings. Although residual correlations were not allowed in the final model, the non-trivial covariances should be critically re-examined in future research to test for recurring misspecification or mere sampling error. Future investigations should also seek to establish test-retest reliability and measurement invariance across gender and age groups, as this was beyond the scope of the present study. So far, we cannot provide evidence that the PPLQ functions equivalently across the heterogeneous group of adults (aged 18–65 years) for whom it was developed, ensuring that any observed differences are due to the underlying construct being measured. In addition, the self-reported nature of the PPLQ presents a limitation per se as it makes the instrument susceptible to recall and social desirability bias. Associations with objective measurements in the physical domain could enhance the validity and explanatory power. The convergent validity comparisons with the PAHCO did not reach acceptable levels for knowledge, understanding and PA behavior, which should be thoroughly readdressed and cross-validated with closely related instruments for the corresponding domain. Finally, unlike to previous studies assessing PL (e.g.,^73^^,^^81^^,^^82^), we did not consider a social domain for operationalization of PL, since it is not a key attribute of the IPLA definition.^6^

## 5 Conclusion

The present study provides first evidence of reliability and validity of the PPLQ, as an instrument to measure PL and its domains in a general adult population in German speaking countries. Compared to other PL instruments for adults, the PPLQ offers a comprehensive measurement model of all essential attributes according to the PL definition of the IPLA,^6^ with the domains being distinct from each other and, therefore, allowing for independent meaningful interpretation. Moreover, the six domains derived from the model can be combined to calculate an overall PL score facilitating the quantification of an individual's ‘PL-level’. This renders the PPLQ a valuable instrument for researchers, but also for practitioners and trainers for charting domain and overall PL levels over time to provide feedback for individuals regarding their progress along their PL journey. However, caution is warranted since specific items, especially the knowledge domain needs to be refined and perhaps approached through formative indicators in further studies. Future large-scale studies with well-stratified samples should also investigate whether the PPLQ model remains invariant across the diverse adult population.

## Funding

This research did not receive any specific external grant from funding agencies in the public, commercial, or not-for-profit sectors.

## Authors' contributions

Conceptualization: Peter Holler & Johannes Jaunig; Data curation: Peter Holler & Johannes Jaunig; Formal analysis: Peter Holler & Johannes Jaunig; Investigation: Peter Holler; Methodology: Peter Holler, Johannes Jaunig & Johannes Carl; Project administration: Peter Holler & Johannes Jaunig, Software: Johannes Jaunig; Visualization: Peter Holler; Supervision: Johannes Jaunig & Mireille N. M. van Poppel. Writing - original draft: Peter Holler & Johannes Jaunig; Writing - review & editing: All authors.

## Declaration of competing interest

All authors declare that they have no conflicts of interest relevant to the content of this article.

## References

[1] Bailey R. (2022). Defining physical literacy: making sense of a promiscuous concept. Sport Soc.

[2] Edwards L.C., Bryant A.S., Keegan R.J., Morgan K., Jones A.M. (2017). Definitions, foundations and associations of physical literacy: a systematic review. Sports Med.

[3] Whitehead M. (2019). Physical Literacy across the World.

[4] Stevens-Smith D.A. (2016). Physical literacy: getting kids active for life. Strategies.

[5] Whitehead M. (2001). The concept of physical literacy. Eur J Phys Educ.

[6] International Physical Literacy Association (2022). https://www.physical-literacy.org.uk/.

[7] Whitehead M. (2010). Physical Literacy: Throughout the Lifecourse.

[8] Whitehead M. (2013). Defnition of physical literacy and clarifcation of related issues. Bull - Int Counc Sport Sci Phys Educ..

[9] Cairney J., Dudley D., Kwan M., Bulten R., Kriellaars D. (2019). Physical literacy, physical activity and health: toward an evidence-informed conceptual model. Sports Med.

[10] Bandura A. (1977). Self-efficacy: toward a unifying theory of behavioral change. Psychol Rev.

[11] Deci E.L., Ryan R.M. (2013).

[12] Bauman A.E., Reis R.S., Sallis J.F., Wells J.C., Loos R.J., Martin B.W. (2012). Correlates of physical activity: why are some people physically active and others not?. Lancet.

[13] Carl J., Barratt J., Wanner P., Töpfer C., Cairney J., Pfeifer K. (2022). The effectiveness of physical literacy interventions: a systematic review with meta-analysis. Sports Med.

[14] Töpfer C., Jaunig J., Carl J. (2022). Physical Literacy – to be discussed: eine Perspektive aus Sicht der deutschsprachigen Sportwissenschaft. Ger J Exerc Sport Res.

[15] Carl J., Bryant A.S., Edwards L.C. (2023). Physical literacy in Europe: the current state of implementation in research, practice, and policy. J Exerc Sci Fit.

[16] Austria Statistik Körperliche aktivität. Statistik Austria. https://www.statistik.at/statistiken/bevoelkerung-und-soziales/gesundheit/gesundheitsverhalten/koerperliche-aktivitaet.

[17] Cornish K., Fox G., Fyfe T., Koopmans E., Pousette A., Pelletier C.A. (2020). Understanding physical literacy in the context of health: a rapid scoping review. BMC Publ Health.

[18] Jones G.R., Stathokostas L., Young B.W. (2018). Development of a physical literacy model for older adults – a consensus process by the collaborative working group on physical literacy for older Canadians. BMC Geriatr.

[19] Petrusevski C., Morgan A., MacDermid J., Wilson M., Richardson J. (2022). Framing physical literacy for aging adults: an integrative review. Disabil Rehabil.

[20] Edwards L.C., Bryant A.S., Keegan R.J., Morgan K., Cooper S.M., Jones A.M. (2018). “Measuring” physical literacy and related constructs: a systematic review of empirical findings. Sports Med.

[21] Kwan M.Y.W., Graham J.D., Bedard C., Bremer E., Healey C., Cairney J. (2019). Examining the effectiveness of a pilot physical literacy–based intervention targeting first-year university students: the PLUS program. Sage Open.

[22] She X., Gao T.Y., Ma R.S., Tang D., Zhong H., Dong H.L. Relationship among positive self-esteem, physical literacy, and physical activity in college students: a study of a mediation model. Front Psychol. 2023;14. 10.3389/fpsyg.2023.1097335. [Accessed 1 September 2023]. PMC10230059 37265948

[23] Barnett L.M., Jerebine A., Keegan R. (2023). Validity, reliability, and feasibility of physical literacy assessments designed for school children: a systematic review. Sports Med.

[24] Dudley D., Cairney J., Wainwright N., Kriellaars D., Mitchell D. (2017). Critical considerations for physical literacy policy in public health, recreation, sport, and education agencies. Quest.

[25] Taplin L. (2011). Physical literacy: an introduction to the concept. Phys Educ Matters.

[26] Young L., O'Connor J., Alfrey L. (2020). Physical literacy: a concept analysis. Sport Educ Soc.

[27] Tremblay M., Lloyd M. (2010). Physical literacy measurement - the missing piece. Phys Health Educ J.

[28] Boldovskaia A., Dias N.M.G., Silva M.N., Carraça E.V. (2023). Physical literacy assessment in adults: a systematic review. PLoS One.

[29] Vašíčková J., Cuberek R., Pernicová H. (2020). Reliabilita Dotazníku sebehodnocení pohybové gramotnosti u vysokoškolské populace. Tělesná Kult.

[30] Ma R.S., Ng S.I., Lee T., Yang Y.J., Sum R.K.W. (2022). Validation of a speech database for assessing college students' physical competence under the concept of physical literacy. Int J Environ Res Publ Health.

[31] Luo L., Song N., Huang J. (2022). Validity evaluation of the college student physical literacy questionnaire. Front Public Health.

[32] Sum R.K.W., Ha A.S.C., Cheng C.F. (2016). Construction and validation of a perceived physical literacy instrument for physical education teachers. PLoS One.

[33] Ma R.S., Sum R.K.W., Hu Y.N., Gao T.Y. (2020). Assessing factor structure of the simplified Chinese version of perceived physical literacy instrument for undergraduates in mainland China. J Exerc Sci Fit.

[34] Mendoza-Muñoz M., Carlos-Vivas J., Castillo-Paredes A., Sum R.K.W., Rojo-Ramos J., Pastor-Cisneros R. (2023). Translation, cultural adaptation and validation of perceived physical literacy instrument-Spanish version (PPLI-Sp) for adults. J Sports Sci Med.

[35] Munusturlar S., Yıldızer G. (2019). Assessing factor structure of perceived physical literacy scale for physical education teachers for Turkish sample. Hacet Univ J Educ Adv Online Publ.

[36] Samadi H., Moradi J., Aghababa A.R. (2022). Psychometric properties of Persian version of the perceived physical literacy instrument (PPLI). Mot Behav.

[37] Gunnell K.E., Longmuir P.E., Barnes J.D., Belanger K., Tremblay M.S. (2018). Refining the Canadian Assessment of Physical Literacy based on theory and factor analyses. BMC Publ Health.

[38] Corbin C.B. (2016). Implications of physical literacy for research and practice: a commentary. Res Q Exerc Sport.

[39] Kilpatrick M., Hebert E., Jacobsen D. (2002). Physical activity motivation: a practitioner's guide to self-determination theory. J Phys Educ Recreat Dance.

[40] Williams S.L., French D.P. (2011). What are the most effective intervention techniques for changing physical activity self-efficacy and physical activity behaviour—and are they the same?. Health Educ Res.

[41] capito Easy-to-understand language and barrier-free informationeichte Sprache - begriffe, Regeln und Beispiele. capito - leichte Sprache. https://www.capito.eu/leichte-sprache/.

[42] Holler P., Jaunig J., Moser O. (2021). Primary care and physical literacy: a non-randomized controlled pilot study to combat the high prevalence of physically inactive adults in Austria. Int J Environ Res Publ Health.

[43] Holler P., Jaunig J., Amort F.M. (2019). Holistic physical exercise training improves physical literacy among physically inactive adults: a pilot intervention study. BMC Publ Health.

[44] Jean de Dieu H., Zhou K. (2021). Physical literacy assessment tools: a systematic literature review for why, what, who, and how. Int J Environ Res Publ Health.

[45] Barnett L., Dudley D.A., Telford R D. (2019). Guidelines for the selection of physical literacy measures in physical education in Australia. J Teach Phys Educ.

[46] Widaman K.F., Little T.D., Preacher K.J., Sawalani G.M., Trzesniewski K.H., Donnellan M.B., Lucas R.E. (2011). Secondary Data Analysis: An Introduction for Psychologists.

[47] Zamanzadeh V., Ghahramanian A., Rassouli M., Abbaszadeh A., Alavi-Majd H., Nikanfar A.R. (2015). Design and implementation content validity study: development of an instrument for measuring patient-centered communication. J Caring Sci.

[48] Lynn M.R. (1986). Determination and quantification of content validity. Nurs Res.

[49] Balza J.S., Cusatis R., McDonnell S.M., Basir M.A., Flynn K.E. (2022). Effective questionnaire design: how to use cognitive interviews to refine questionnaire items. J Neonatal Perinat Med.

[50] Strauss A.L., Corbin J.M. (1998).

[51] Carl J., Sudeck G., Geidl W., Schultz K., Pfeifer K. (2021). Competencies for a healthy physically active lifestyle—validation of an integrative model. Res Q Exerc Sport.

[52] Carl J., Sudeck G., Pfeifer K. (2020). Competencies for a healthy physically active lifestyle: second-order analysis and multidimensional scaling. Front Psychol.

[53] FH JOANNEUM MOVEluencer. https://www.fh-joanneum.at/projekt/moveluencer/.

[54] Ekelund U., Tarp J., Steene-Johannessen J. (2019). Dose-response associations between accelerometry measured physical activity and sedentary time and all cause mortality: systematic review and harmonised meta-analysis. BMJ.

[55] Moore S.C., Patel A.V., Matthews C.E. (2012). Leisure time physical activity of moderate to vigorous intensity and mortality: a large pooled cohort analysis. PLoS Med.

[56] Arem H., Moore S.C., Patel A. (2015). Leisure time physical activity and mortality: a detailed pooled analysis of the dose-response relationship. JAMA Intern Med.

[57] Carl J., Sudeck G., Pfeifer K. (2020). Competencies for a healthy physically active lifestyle—reflections on the model of physical activity-related health competence. J Phys Activ Health.

[58] Dray S., Josse J. (2015). Principal component analysis with missing values: a comparative survey of methods. Plant Ecol.

[59] Terwee C.B., Mokkink L.B., van Poppel M.N.M., Chinapaw M.J.M., van Mechelen W., de Vet H.C.W. (2010). Qualitative attributes and measurement properties of physical activity questionnaires. Sports Med.

[60] Hu L., Bentler P.M. (1999). Cutoff criteria for fit indexes in covariance structure analysis: conventional criteria versus new alternatives. Struct Equ Model Multidiscip J.

[61] Xia Y., Yang Y. (2019). RMSEA, CFI, and TLI in structural equation modeling with ordered categorical data: the story they tell depends on the estimation methods. Behav Res Methods.

[62] Savalei V. (2021). Improving fit indices in structural equation modeling with categorical data. Multivariate Behav Res.

[63] Schermelleh-Engel K., Moosbrugger H., Müller H. (2003). Evaluating the fit of structural equation models: tests of significance and descriptive goodness-of-fit measures. Methods Psychol Res.

[64] Awang Z. (2015).

[65] Awang P.D.Z. (2018). https://eprints.unisza.edu.my/3865/.

[66] Lawshe C.H.A. (1975). Quantitative approach to content Validity1. Person Psychol.

[67] Fornell C., Larcker D.F. (1981). Evaluating structural equation models with unobservable variables and measurement error. J Mar Res.

[68] R Core Team. R (2022).

[69] Rosseel Y. (2012). Lavaan: an R package for structural equation modeling. J Stat Software.

[70] Jorgensen Terrence D. Package ‘semTools.’. https://cran.r-project.org/web/packages/semTools/semTools.pdf.

[71] Farooq R. (2022). Heywood cases: possible causes and solutions. Int J Data Anal Tech Strat.

[72] Mota J., Martins J., Onofre M. (2021). Portuguese Physical Literacy Assessment Questionnaire (PPLA-Q) for adolescents (15–18 years) from grades 10–12: development, content validation and pilot testing. BMC Publ Health.

[73] Mota J., Martins J., Onofre M., Dudley D. Portuguese Physical Literacy Assessment for adolescents (15–18 years): validation using confirmatory factor and composite analyses. Front Sports Act Living. 2023;5. 10.3389/fspor.2023.1192025. [Accessed 9 September 2023]. 10.3389/fspor.2023.1192025 PMC10333539 37440875

[74] Mota J., Martins J., Onofre M. (2023). Portuguese physical literacy assessment questionnaire (PPLA-Q) for adolescents: validity and reliability of the psychological and social modules using mokken scale analysis. Percept Mot Skills.

[75] Edwards L.C., Bryant A.S., Keegan R.J., Morgan K., Jones A.M. (2017). Definitions, foundations and associations of physical literacy: a systematic review. Sports Med.

[76] Cale L., Harris J. (2018). The role of knowledge and understanding in fostering physical literacy. J Teach Phys Educ.

[77] Ryom K., Hargaard A.S., Melby P.S. (2022). Self-reported measurements of physical literacy in adults: a scoping review. BMJ Open.

[78] Kline R.B. (2015).

[79] Britton Ú., Belton S., Peers C. (2023). Physical literacy in children: exploring the construct validity of a multidimensional physical literacy construct. Eur Phys Educ Rev.

[80] Longmuir P.E., Woodruff S.J., Boyer C., Lloyd M., Tremblay M.S. (2018). Physical Literacy Knowledge Questionnaire: feasibility, validity, and reliability for Canadian children aged 8 to 12 years. BMC Publ Health.

[81] Allan V., Turnnidge J., Côté J. (2017). Evaluating approaches to physical literacy through the lens of positive youth development. Quest.

[82] Barnett L.M., Mazzoli E., Bowe S.J., Lander N., Salmon J. (2022). Reliability and validity of the PL-C Quest, a scale designed to assess children's self-reported physical literacy. Psychol Sport Exerc.

