# Supplementary material for: Development of the Perceived Physical Literacy Questionnaire (PPLQ) for the adult population
Source: J Exerc Sci Fit. 2023 Oct 5;21(4):424–33. doi: 10.1016/j.jesf.2023.09.003 (PMC10661355; doi:10.1016/j.jesf.2023.09.003)
Supplement: Multimedia component 1 [file mmc1.docx]

**Appendix A**

**Table of content:**

- Stage 1: pages 2 to 4
- Stage 2: pages 5 to 13
- Stage 3: pages 13 to 15
- Stage 4: pages 16 to 21
- Stage 5: page 22

**A1. Stage 1**

**A1.1. Methods: Stage 1**

To revise our former PL instrument,^1,2^ a literature review following a selective search approach was conducted on PubMed, ScienceDirect and Google Scholar during October 2019. The aim in stage 1 was to generate a large item pool from established questionnaires (subscales) for our underlying PL domains, preferably validated in German language. The applied keywords presented each a combination of the term “questionnaire” and the name of the respective domain, whereby also the synonyms of the keywords were considered (e.g., scale, tool, and instrument for the term “questionnaire”). The keywords were entered in German and English language. Where appropriate, the questionnaires (subscales) from our previous instrument were retained. All changes were discussed between the first (PH) and last author (JJ) of this manuscript to achieve consensus regarding the inclusion into the item pool.

**A1.2. Results: Stage 1**

Table A.1. presents the results regarding the final selected questionnaire (subscales) and items in the revised questionnaire from stage 1 (referred as PPLQ version 1). We could not identify any established questionnaires (subscales) for the domain understanding and knowledge fitting the PL framework, and the subscales in our former questionnaire were found to be inappropriate (i.e., some items in the understanding domain were thematically focused on motivation and confidence, and the knowledge domain was operationalized by open-ended questions). Therefore, we additionally adjusted item wordings and self-constructed new items for these domains. Overall, the questionnaire included 61 items.

**Table A.1.** Revised questionnaire at the end of stage 1 (PPLQ version 1)

| Domain  **Table A.1:** Revised uestionnaire at the end of stage | Measurement |
| --- | --- |
| **Physical competence**  4 subfactors:   - strength - endurance - coordination - sport competence | **Physical Self-Description Questionnaire - Short Version (PSDQ-S)**  Dimensionality of the domain 🡪 four subscales: (i) endurance (3 items), (ii) strength (3 items), (iii) coordination (3 items), (iv) sport competence subscales (5 items)^3,4^  **Strength subscale:**   1. *“I am a physically strong person”* 2. “*I would do well in a test of strength”* 3. *“I have a lot of power in my body”*   **Endurance subscale:**   1. “*I can be physically active for a long period of time without getting tired”* 2. *“I think I could run a long way without getting tired”* 3. *“I am good at endurance activities e.g., distance running, aerobics, cycling, swimming or cross-country skiing”*   **Coordination subscale:**   1. “*I feel confident in performing difficult and rapid successive movements”* 2. *“I find it easy to control the movements of my body”* 3. *“I can coordinate my movements well”* 4. *“In most physical activities, my movements are smooth and even”* 5. *“I find my body can make steady movements easily”*   **Sport competence subscale:**   1. *“I'm good at most sports”* 2. *“I have good sporting skills”* 3. *“I'm good at sports games”*   **Response scale (for all items):**  Likert scale from 1 (= strongly disagree) to 6 (= strongly agree) |

| Domain  **Table A.1:** Revised uestionnaire at the end of stage | Measurement |
| --- | --- |
| **Understanding** | Six out of seven items in total were adapted from Fitzgerald et al. (1994)^5^, Sum et al. (2018)^6^, Brooks et al. (2018, 2 items)^7^ and Holler et al. (2019; 2 items)^1^ respectively. One item was self-constructed. Dimensionality of the domain: Due to the lack of a theory, we assumed the domain to be one-dimensional.  **Items:**   1. *“If I have a choice, I tend to use my car rather than my bike for shorter distances”* ^(adapted from Fitzgerald et al., 1994)^ 2. *“I feel less appreciation for others engaging in regular PA”* ^(adapted from Sum et al., 2018)^ 3. *“I don’t see why I should bother to do PA”* ^(adapted from Brooks et al., 2018)^ 4. *“I think engaging in PA regularly is a waste of time”* ^(adapted from Brooks et al., 2018)^ 5. *“I think regular sport lessons at school are unnecessary”* ^(adapted from Holler et al., 2019)^ 6. *“If I have a choice, I use the elevator and/or escalator rather than the stairs”* ^(adapted from Holler et al., 2019)^ 7. *“I consider initiatives in companies or organizations to increase PA (e.g., company walking day) to be superfluous”* ^(self-constructed)^   **Response scale (for all items):**  Likert scale from 1 (= strongly disagree) to 6 (= strongly agree) |
| **Motivation**  4 subfactors:   - intrinsic motivation - identified motivation - introjected motivation - extrinsic motivation | **Sport-and Exercise-Related Self-Concordance-Scale (SSK-Scale)**  Dimensionality of the domain 🡪 four subscales: (i) intrinsic (3 items), (ii) identified (3 items), (iii) introjected (3 items) and (iv) extrinsic motivation subscales (3 items)^8^  **Introductory sentence (prefaced stem to all items):**  *“I intend to be regularly active in sports in the coming weeks and months, …”*  **Intrinsic motivation subscale:**   1. *“… because I simply enjoy it”* 2. *“…because it gives me experiences that I wouldn't want to miss”* 3. *“…because sporting activity is simply part of my life”*   **Identified motivation subscale:**   1. *“…because I have good reasons for it”* 2. *“…because it is good for me”* 3. *“…because the positive consequences are simply worth the effort”*   **Introjected motivation subscale:**   1. *“…because otherwise I would have a guilty conscience”* 2. *“…because I think that sometimes you also have to force yourself to do something”* 3. *“…because otherwise I would have to reproach myself”*   **Extrinsic motivation subscale:**   1. *“…because people who are important to me urge me to do so”* 2. *“…because otherwise I get into trouble with other people”* 3. *“…because others say I should be active in sports”*   **Response scale (for all items):**  Likert scale from 1 (= strongly disagree) to 6 (= strongly agree) |
| **Confidence (i.e., self-efficacy)** | **Self-efficacy for sports activity scale (SSA scale, 12 items)**^9^  Dimensionality of the domain: The scale is one-dimensional.  **Introductory sentence (prefaced stem to all items):**  “*I am sure that I will still be able to perform a planned sports activity even if..*.”  **Items:**   1. *“... I am tired”* 2. *“… I feel depressed”* 3. *“… I have worries”* 4. *“… I am annoyed about something”* 5. “…*I feel tense”* 6. *“… friends are there for a visit”* 7. *“…my family/partner makes demands on me”* 8. *“…other people want to do something with me”* 9. *“... I can't find anyone to do sports with me”* 10. “... *the weather is bad*” 11. “… *I still have a lot of work to do*” 12. *“… an interesting TV program is running”*   **Response scale (for all items):**  Likert scale from 1 (= strongly disagree) to 6 (= strongly agree) |

**Table A.1 (continued):** Revised questionnaire at the end of stage 1 (PPLQ version 1)

| Domain | Measurement |
| --- | --- |
| **PA Behavior** | **International Physical Activity Questionnaire - Short Form (IPAQ-SF)**^10^  Dimensionality of the domain: Not applicable since PA behavior is not operationalized by a reflective measurement model.  **Description:**  The IPAQ-SF is an internationally accepted and validated self-report questionnaire to evaluate the PA status of persons aged between 15 and 69 years by recording the number of days and time (hours/min) spent on PA in moderate intensity, vigorous intensity and walking for at least 10 minutes as well as time spent sitting during the last 7 days. |
| **Knowledge** | Four out of nine items in total were adapted from Morrow et al. (2004; 2 items)^11^, Hui and Morrow (2001)^12^ and Rowland et al. (1994)^13^. Five items were self-constructed. Dimensionality of the domain: Following a theory-driven approach, the items were adapted/constructed to be ground into two subscales, how to move  (item i, v, vi, vii, & viii) and knowledge of benefits (item ii, iii & ix; see below for a rationale of the two-factor-structure).  **Items** (correct answers in bold)   1. *“According to the Austrian PA guidelines, at least how many minutes per week should you perform endurance-oriented activities that involve a slight increase in breathing and pulse rate (e.g., brisk walking)?” [30 min, 45 min, 60 min, 75 min, 90 min, 120 min,* ***150 min****, 180 min, 240 min]* ^(adapted from Morrow et al., 2004)^ 2. *“A physically inactive lifestyle increases the risk of suffering the following diseases” [****breast cancer, dementia, hypertension****]* ^(adapted from Hui and Morrow, 2001)^ 3. *“PA can improve the course of the following diseases” [****sugar disease (diabetes mellitus type II) Parkinson's disease, joint wear (arthrosis)****] ^(^*^adapted from Roland et al., 1994)^ 4. *“Three 10-minutes sessions of PA three times per week provide the same health benefits as a single 30-minutes-sessions” [****true****/false]* ^(adapted from Morrow et al., 2004)^ 5. *“Strength can only be trained up to the age of 60” [true,* ***false****]* ^(self-constructed)^ 6. *“Strength training alone (without endurance training) does not result in any health benefits” [true,* ***false****]* ^(self-constructed)^ 7. *“Women need different strength exercises than men to build muscle” [true,* ***false****]* ^(self-constructed)^ 8. *“Strength training is not suitable for losing weight (body fat)” [true,* ***false****]* ^(self-constructed)^ 9. *“A person (80kg) consumes how many kilocalories per hour (approximately) through normal walking” [50 kcal, 100 kcal,* ***300 kcal****, 500 kcal, 800 kcal, 1000 kcal]* ^(self-constructed)^   **Rationale of the two-factor-structure of the knowledge domain:**  In most studies on PL, the construct knowledge is combined with understanding into a single domain that encompasses three components: (i) knowledge and understanding of physical/sport activities, (ii) knowledge and understanding of a healthy physically active lifestyle and (iii) the value to take responsibility for PA.^14^ However, when considering knowledge as a separate domain, we believed that only the first two components correspond to this domain, as they are clearly cognitive in nature. In our opinion, the latter component refers more to a person's attitude towards PA and therefore reflects more the notion of understanding, which is presented as a separate domain in our hypothetical PL model. Consequently, for the operationalization of knowledge, we only referred to the first two components mentioned above. For brevity, we labeled them as "how to move" and "knowledge of benefits". |

**Table A.1 (continued):** Revised questionnaire at the end of stage 1 (PPLQ version 1)

**A2. Stage 2**

**A.2.1. Methods: Stage 2**

In stage 2 we aimed to identify the best fitting items from the generated item pool in stage 1. For the item reduction procedure our premises were to have a maximum of two subscales per domain with a minimum of three items within a subscale. For this aim, a cross-sectional study was conducted in November 2019, in which students and university staff members were asked to complete an online version of the PPLQ version 1. Following a convenience sampling approach, participants were recruited via an open call for participation distributed via mailing lists at the University of Graz and the FH JOANNEUM - University of Applied Sciences (both universities are in Styria, a province of Austria). To take part in the survey, participants had to be between 18 and 65 years and speak German fluently. A total of 598 individuals completed the questionnaire, whereby 92 were excluded due to incomplete answers (i.e., left the survey before completion). The final sample consisted of 506 participants (72% female), with a mean age of 27.31 ± 10.11 years.

The subsequent item reduction procedure varied per domain and is therefore described in detail together with the corresponding results. Overall, we followed a two-step-approach in each case. In a first step, an exploratory factor analysis (EFA) using principal component analysis with varimax rotation was performed for each domain, except for PA behavior as it is not operationalized by a reflective measurement model. Only factors with eigenvalues greater than 1.0 were retained. Items loading of .50 and above on the factor of interest were considered as adequate. Cross-loadings were defined as an item with loadings of at least .40 on two or more factors. Additional to the EFA, internal consistency was assessed using Cronbach’s alpha. The subscales/items with the most appropriate measurement properties (i.e., factor loadings and Cronbach's alpha coefficients) were selected.^15^ All statistical analyses were conducted using IBM SPSS 25. In a second step, the obtained results were critically discussed with seven experts (three senior and four early-stage researchers all working in the field of PA and/or public health, all familiar with the PL concept). If applicable, the content validity index (CVI) was used, a method to calculate content validity quantitatively.^16^ Applying the CVI, the experts were asked to give ratings of “relevant (1)” or “non relevant (0)” to all subscales/items within one domain. The CVI was subsequently calculated as the number of experts giving a rating of “relevant” for each item/subscale divided by the total number of experts. CVI-values ranged from 0 to 1, with a CVI of at least 0.83 (six out of seven) indicating satisfactory content validity.^17^

**A.2.2. Results: Stage 2**

**Domain: Physical competence**

An EFA including the four subscales from the domain physical competence revealed a three-factor structure (see Table A.2), with an explained variance of 74.28%. The Bartlett test reached statistical significance (p < .001) and the Kaiser-Meyer-Olkin (KMO) criterion was .93, which suggested that the data was very suitable for factor analysis. Only the three items from the sport competence subscale (SP1, SP2 and SP3) was found to be inadequate (i.e., low factor and cross loadings), which forced us to exclude this subscale. An EFA on the remaining items revealed an adequate factor loading pattern in the hypothesized direction for the strength, endurance, and coordination subscale (date not shown). Additionally, Cronbach’s alpha coefficients indicated a high internal consistency for all three factors, with .93 for the strength, .85 for the endurance and .89 for the coordination subscale, respectively.

Following our premise of having a maximum of two subscales per domain, in a second step we aimed to excluded one more subscale based on an expert rating. For this purpose, the seven experts were asked to rate the remaining three subscales regarding their relevance for a PL questionnaire for adults, either as “relevant (x)” or “non relevant (-)”. Besides, they were asked to justify their answers briefly (open-end question). The results from the expert rating are illustrated in Table A.3.

**Table A.2:** Factor matrix of four subscales from the PSDQ-S after varimax rotation

| Items | | Factorial loadings | | | |
| --- | --- | --- | --- | --- | --- |
|  | **Strength subscale** |  |  |  |  |
| ST1 | I am a physically strong person | .845 |  |  |  |
| ST2 | I would do well in a test of strength | .838 |  |  |  |
| ST3 | I have a lot of power in my body | .877 |  |  |  |
|  | **Endurance subscale** |  |  |  |  |
| EN1 | I can be physically active for a long period of time without getting tired |  | .778 |  |  |
| EN2 | I think I could run a long way without getting tired |  | .631 |  |  |
| EN3 | I am good at endurance activities e.g., distance run, aerobics, cycling, swim or cross-country ski |  | .890 |  |  |
|  | **Coordination subscale** |  |  |  |  |
| CO1 | I feel confident in performing difficult and rapid successive movements |  |  | .629 |  |
| CO2 | I find it easy to control the movements of my body |  |  | .780 |  |
| CO3 | I can coordinate my movements well |  |  | .825 |  |
| CO4 | In most physical activities, my movements are smooth and even |  |  | .795 |  |
| CO5 | I find my body can make steady movements easily |  |  | .787 |  |
|  | **Sport subscale** |  |  |  |  |
| SP1 | I'm good at most sports |  |  |  | .749 |
| SP2 | I have good sporting skills | .504 |  |  | .568 |
| SP3 | I'm good at sports games |  |  |  | .888 |

**Table A.2:** Factor matrix of four selected subscales from the PSDQ-S after Varimax rotation

| Items | | Factor loadings | | |
| --- | --- | --- | --- | --- |
|  | **Strength subscale** |  |  |  |
| ST1 | I am a physically strong person | .850 |  |  |
| ST2 | I would do well in a test of strength | .850 |  |  |
| ST3 | I have a lot of power in my body | .887 |  |  |
|  | **Endurance subscale** |  |  |  |
| EN1 | I can be physically active for a long period of time without getting tired |  | .800 |  |
| EN2 | I think I could run a long way without getting tired |  | .645 |  |
| EN3 | I am good at endurance activities e.g., distance running, aerobics, cycling, swimming or cross-country skiing” |  | .876 |  |
|  | **Coordination subscale** |  |  |  |
| CO1 | I feel confident in performing difficult and rapid successive movements |  |  | .649 |
| CO2 | I find it easy to control the movements of my body |  |  | .778 |
| CO3 | I can coordinate my movements well |  |  | .842 |
| CO4 | In most physical activities, my movements are smooth and even |  |  | .793 |
| CO5 | I find my body can make steady movements easily |  |  | .799 |
|  | **Sport subscale** |  |  |  |
| SP1 | I'm good at most sports |  | .462 | .559 |
| SP2 | I have good sporting skills | .428 | .477 | .608 |
| SP3 | I'm good at sports games |  | .433 | .423 |

| Subscales | Experts  **Table A.3:** Expert ratings on the relevance of the physical competence subscales  **Table A.3:** Expert ratings on the relevance of the physical competence subscales | | | | | | | CVI |
| --- | --- | --- | --- | --- | --- | --- | --- | --- |
|  | 1 | 2 | 3 | 4 | 5 | 6 | 7 |  |
| Strength | x | x | - | x | x | x | x | 0.86 |
| Endurance | x | x | x | x | x | x | x | 1.00 |
| Coordination | - | - | x | - | - | - | - | 0.14 |

The two subscales strength and endurance reach an adequate level of relevance within the expert rating, with a CVI of 0.86 (six out of seven) and 1.00 (seven out of seven), respectively. Against this background only these two subscales (with all corresponding items, three items each) were included as measure of physical competence in the final PPLQ version 2. Running an EFA on the six items from these two subscales, the two-factor structure could be confirmed (84.4% of the variance explained; Bartlett test p < .001; KMO criterion of .85). All factor loadings were over 0.5 (range between .632 and .882), with no cross loading detected.

Rational for the selection of the two subscales (summary of the expert’s statements):

All experts declared that generally all three subscales are of relevance, since within the PL framework, physical competence refers to an individual’s ability to move with competence in a wide variety of activities.^14^ However, when age is included, the relevance of these three components shifts. They argued that coordination might be more important for children and adolescents to master a wide variety of movements/sport skills, while with increasing age the maintenance and development of an adequate level of endurance and strength becomes paramount.^18^ These two components are considered as obligatory for a majority of daily activities such as walking, climbing stairs, or cycling.^19^ In lights of this, two experts emphasized that having a limited level of strength and endurance in adulthood is tantamount to a standstill of a person’s PL journey. Most of the experts (five out of seven) also refer in their justifications to the national and international PA guidelines,^20,21^ which rather emphasize the importance of endurance and strength among adults and elderly then coordination.

**Domain: Understanding**

Contrary to our assumption that the domain understand is one-dimensional, an EFA revealed a two-factor solution (see table A.4), with 51.5% of the variance explained. The Bartlett test was significant (p < .001) with a KMO criterion of .76. No item loadings below .50 and substantial cross loading were observed. However, only two items loaded on the second factor, which contradicted our premise of at least three items per subscale. As a result, we decided to exclude the second factor with the corresponding item in this first step.

**Table A.4:** Factor matrix of the domain understanding after varimax rotation

| Items | | Factorial loadings | | | |
| --- | --- | --- | --- | --- | --- |
|  | **Strength subscale** |  |  |  |  |
| ST1 | I am a physically strong person | .845 |  |  |  |
| ST2 | I would do well in a test of strength | .838 |  |  |  |
| ST3 | I have a lot of power in my body | .877 |  |  |  |
|  | **Endurance subscale** |  |  |  |  |
| EN1 | I can be physically active for a long period of time without getting tired |  | .778 |  |  |
| EN2 | I think I could run a long way without getting tired |  | .631 |  |  |
| EN3 | I am good at endurance activities e.g., distance run, aerobics, cycling, swim or cross-country ski |  | .890 |  |  |
|  | **Coordination subscale** |  |  |  |  |
| CO1 | I feel confident in performing difficult and rapid successive movements |  |  | .629 |  |
| CO2 | I find it easy to control the movements of my body |  |  | .780 |  |
| CO3 | I can coordinate my movements well |  |  | .825 |  |
| CO4 | In most physical activities, my movements are smooth and even |  |  | .795 |  |
| CO5 | I find my body can make steady movements easily |  |  | .787 |  |
|  | **Sport subscale** |  |  |  |  |
| SP1 | I'm good at most sports |  |  |  | .749 |
| SP2 | I have good sporting skills | .504 |  |  | .568 |
| SP3 | I'm good at sports games |  |  |  | .888 |

**Table A.2:** Factor matrix of four selected subscales from the PSDQ-S after Varimax rotation

|  | Items | Factor loadings | |
| --- | --- | --- | --- |
| UN1 | If I have a choice, I tend to use my car rather than my bike for shorter distances |  | .698 |
| UN2 | I feel less appreciation for others engaging in regular PA | .561 |  |
| UN3 | I don’t see why I should bother to do PA | .742 |  |
| UN4 | I think engaging in PA regularly is a waste of time | .779 |  |
| UN5 | I think regular sport lessons at school are unnecessary | .656 |  |
| UN6 | If I have a choice, I use the elevator and/or escalator rather than the stairs |  | .652 |
| UN7 | I consider initiatives in companies or organizations to increase PA (e.g., company walking day) to be superfluous | .631 |  |

In a second step, the results were presented to the seven experts. To identify further opportunities for an item reduction, they were asked to rate the remaining five items from the first factor either as “relevant (x)” or “non relevant (-)”. Besides, they were asked to justify their answers briefly (open-end question). The results from the expert rating are illustrated in Table A.5.

**Table A.5:** Expert ratings on the relevance of the items within the domain understanding

**Table A.3:** Expert ratings on the relevance of the physical competence subscales

| Subscales | Experts | | | | | | | CVI |
| --- | --- | --- | --- | --- | --- | --- | --- | --- |
|  | 1 | 2 | 3 | 4 | 5 | 6 | 7 |  |
| UN2 | x | x | x | - | x | x | x | 0.86 |
| UN3 | - | x | - | - | - | - | x | 0.29 |
| UN4 | x | x | x | x | x | x | - | 0.86 |
| UN5 | x | - | - | - | - | - | - | 0.14 |
| UN7 | x | - | x | - | x | - | x | 0.57 |

Only the two items UN2 and UN4 reach an adequate level of relevance within the expert rating, with a CVI of 0.86 each (six out of seven). Since we aimed to have at least three items per (sub-)scale, we also included item UN7 as measure of understanding in the final PPLQ version 2. The item UN3 and UN5 were excluded. An EFA including the remaining three items confirmed a one-factor solution with an adequate level of explained variance of 55.1%. The Bartlett test was significant (p < .001) with a borderline KMO criterion of .62. The factor loadings were in an adequate range between .682 and .772. However, the Cronbach’s alpha coefficient was .55, which indicated a poor internal consistency of this scale.

Rational for the selection of the three items (summary of the expert’s statements):

Overall, the experts indicated that they designed their rating to ensure heterogeneity in terms of the content of the items. Against this background, they argued that item UN3 was considered less relevant because it was very similar in content to item UN4 but had a lower factor loading in the pairwise comparison. In addition, most experts classified item UN5 as "non relevant" because they believe it could lead to biased responses. They argued that there is a risk that responses to item UN5 could be based on past experiences (i.e., how physical education was experienced in school) rather than current perceptions of its importance per se. Similar comments were made by some experts on item UN7. However, this item was considered as more appropriate for the adulthood population in comparison to the item UN5, since it is more related to the everyday life of adults and the participation in such initiatives is not mandatory compared to physical education.

**Domain: Motivation**

An EFA including the four subscales from the domain motivation revealed a three-factor structure (see Table A.6), with an explained variance of 67.93%. The Bartlett test reached statistical significance (p < .001) and the KMO criterion was .82. No item loading below .50 and relevant cross loadings were observed. Adequate factor loading patterns in the hypothesized direction were evident for the items of introjected and extrinsic motivation subscales, while the six items from the intrinsic and identified motivation subscales loaded only on one factor. The subsequent calculated Cronbach’s alpha coefficients for the three subscales were .86 for intrinsic/identified motivation, .81 for introjected motivation and 0.84 for extrinsic motivation, indicating a high internal consistency for all subscales. Overall, no subscales or items were excluded based on the obtained statistical results.

Following our premise of having a maximum of two subscale per domain, in a second step we aimed to exclude one more subscale and if applicable also items based on an expert rating. For the purpose of exclusion of a subscale, the seven experts were asked to rate the three subscales regarding their relevance for a PL questionnaire for adults, either as “relevant (x)” or “non relevant (-)”. Besides, they were asked to justify their answers briefly (open-end question). The results from the expert rating are illustrated in Table A.7.

| Items | | Factor loadings | | |
| --- | --- | --- | --- | --- |
|  | **Intrinsic motivation subscale** |  |  |  |
| IN1 | … because I simply enjoy it | .880 |  |  |
| IN2 | …because it gives me experiences that I wouldn't want to miss | .877 |  |  |
| IN3 | …because sporting activity is simply part of my life | .782 |  |  |
|  | **Identified motivation subscale** |  |  |  |
| EN1 | …because I have good reasons for it | .729 |  |  |
| EN2 | …because it is good for me | .795 |  |  |
| EN3 | …because the positive consequences are simply worth the effort | .736 |  |  |
|  | **Introjected motivation subscale** |  |  |  |
| IT1 | …because otherwise I would have a guilty conscience |  |  | .748 |
| IT2 | …because I think that sometimes you also have to force yourself to do something |  |  | .774 |
| IT3 | …because otherwise I would have to reproach myself |  |  | .733 |
|  | **Extrinsic motivation subscale** |  |  |  |
| EX1 | …because people who are important to me urge me to do so |  | .850 |  |
| EX2 | …because otherwise I get into trouble with other people |  | .858 |  |
| EX3 | …because others say I should be active in sports |  | .750 |  |

**Table A.6:** Factor matrix of four subscales from the SSK-Scale after varimax rotation

| Items | | Factorial loadings | | | |
| --- | --- | --- | --- | --- | --- |
|  | **Strength subscale** |  |  |  |  |
| ST1 | I am a physically strong person | .845 |  |  |  |
| ST2 | I would do well in a test of strength | .838 |  |  |  |
| ST3 | I have a lot of power in my body | .877 |  |  |  |
|  | **Endurance subscale** |  |  |  |  |
| EN1 | I can be physically active for a long period of time without getting tired |  | .778 |  |  |
| EN2 | I think I could run a long way without getting tired |  | .631 |  |  |
| EN3 | I am good at endurance activities e.g., distance run, aerobics, cycling, swim or cross-country ski |  | .890 |  |  |
|  | **Coordination subscale** |  |  |  |  |
| CO1 | I feel confident in performing difficult and rapid successive movements |  |  | .629 |  |
| CO2 | I find it easy to control the movements of my body |  |  | .780 |  |
| CO3 | I can coordinate my movements well |  |  | .825 |  |
| CO4 | In most physical activities, my movements are smooth and even |  |  | .795 |  |
| CO5 | I find my body can make steady movements easily |  |  | .787 |  |
|  | **Sport subscale** |  |  |  |  |
| SP1 | I'm good at most sports |  |  |  | .749 |
| SP2 | I have good sporting skills | .504 |  |  | .568 |
| SP3 | I'm good at sports games |  |  |  | .888 |

**Table A.2:** Factor matrix of four selected subscales from the PSDQ-S after Varimax rotation

**Table A.7:** Expert ratings on the relevance of the motivation subscales

**Table A.3:** Expert ratings on the relevance of the physical competence subscales

| Subscales | Experts | | | | | | | CVI |
| --- | --- | --- | --- | --- | --- | --- | --- | --- |
|  | 1 | 2 | 3 | 4 | 5 | 6 | 7 |  |
| Intrinsic/identified motivation | x | x | x | x | x | x | x | 1.00 |
| Introjected motivation | x | - | - | x | - | x | - | 0.29 |
| Extrinsic motivation | - | - | - | x | - | - | - | 0.14 |

Only the subscales intrinsic/identified motivation have received an adequate level of relevance, with a CVI of 1.00 (seven out of seven). Against this background, we excluded the subscales introjected and extrinsic motivation. Since this subscale consisted of 6 items, we originally wanted to conduct another rating afterwards to identify further possibilities for an item reduction. However, there was a high degree of consensus among the experts that a two-factor structure might re-emerge in a latter application of the questionnaire and, if so, there would be too few items per factor. Therefore, a further expert rating was not conducted. The subscale with all corresponding six items were included as measure of motivation in the final PPLQ version 2. Running a EFA on the six items from this remaining subscale, a one factor-structure was revealed (65.7% of the variance explained; Bartlett test p < .001; KMO criterion of .85). The factor loadings ranged between .746 and .860, with no cross-loadings observed. An adequate level of internal consistency indicated by a Cronbach’s alpha coefficients of .86 was evident.

Rational for the selection of the two subscales (summary of the expert’s statements):

In their justifications, the experts primarily referred to the International Physical Literacy Association (IPLA) conceptualization of PL, in which motivation is described as a person's enthusiasm and pleasure in embracing PA as an integral part of life.^22^ They argued that this form of motivation implies a very strong sense of autonomy, after which PA is performed out of self-satisfaction, interest, and pleasure in the behavior itself. There was a high degree of consensus, that this form of motivation corresponds best with intrinsic motivation and to some extent also with identified motivation, but less to introjected and extrinsic motivation.^23^ According to these latter forms of motivation, PA is performed because of the reward or prize, which contradicts the International Physical Literacy Association (IPLA) conception of PA motivation.

**Domain: Confidence (i.e., self-efficacy)**

Even though the Self-efficacy for Sports Activity Scale (SSA Scale) is supposed to be one-dimensional, an EFA revealed a three-factor solution (see Table A.8), with 66.9% of the variance explained. The Bartlett test was significant (p < .001) with a KMO criterion of .85. Two items (CO7 and CO11) were observed with a factor loading below .50, while a cross loading was also found for the item CO11. Against this background, these two items were excluded in this first step. An EFA on the remaining 10 items revealed an adequate factor loading pattern. The Cronbach’s alpha coefficients was .87 for the first, .82 for the second and .76 for the third factor, indicating all an adequate level of internal consistency.

|  | Items | Factor loadings | | |
| --- | --- | --- | --- | --- |
| CO1 | ... I am tired | .764 |  |  |
| CO2 | … I feel depressed | .826 |  |  |
| CO3 | … I have worries | .857 |  |  |
| CO4 | … I am annoyed about something |  | .868 |  |
| CO5 | …I feel tense | .778 |  |  |
| CO6 | … friends are there for a visit |  | .810 |  |
| CO7 | …my family/partner makes demands on me |  | .477 |  |
| CO8 | …other people want to do something with me |  | .801 |  |
| CO9 | ... I can't find anyone to do sports with me |  |  | .849 |
| CO10 | ... the weather is bad |  |  | .804 |
| CO11 | … I still have a lot of work to do | .468 | .510 |  |
| CO12 | … an interesting TV program is running |  |  | .711 |

**Table A.8:** Factor matrix of the domain confidence (SSA-Scale) after varimax rotation

| Items | | Factorial loadings | | | |
| --- | --- | --- | --- | --- | --- |
|  | **Strength subscale** |  |  |  |  |
| ST1 | I am a physically strong person | .845 |  |  |  |
| ST2 | I would do well in a test of strength | .838 |  |  |  |
| ST3 | I have a lot of power in my body | .877 |  |  |  |
|  | **Endurance subscale** |  |  |  |  |
| EN1 | I can be physically active for a long period of time without getting tired |  | .778 |  |  |
| EN2 | I think I could run a long way without getting tired |  | .631 |  |  |
| EN3 | I am good at endurance activities e.g., distance run, aerobics, cycling, swim or cross-country ski |  | .890 |  |  |
|  | **Coordination subscale** |  |  |  |  |
| CO1 | I feel confident in performing difficult and rapid successive movements |  |  | .629 |  |
| CO2 | I find it easy to control the movements of my body |  |  | .780 |  |
| CO3 | I can coordinate my movements well |  |  | .825 |  |
| CO4 | In most physical activities, my movements are smooth and even |  |  | .795 |  |
| CO5 | I find my body can make steady movements easily |  |  | .787 |  |
|  | **Sport subscale** |  |  |  |  |
| SP1 | I'm good at most sports |  |  |  | .749 |
| SP2 | I have good sporting skills | .504 |  |  | .568 |
| SP3 | I'm good at sports games |  |  |  | .888 |

**Table A.2:** Factor matrix of four selected subscales from the PSDQ-S after Varimax rotation

In a second step, the obtained results were critically discussed with the seven experts. Following our premise of having a maximum of two subscale per domain, we aimed to exclude at least one factor. However, since not all three factors could be interpreted meaningfully in terms of content, we decided that a quantitative rating is not appropriate at this point. As a result of the discussion with the expert committee, only the first and third factor allowed a reasonable interpretation as internal and external barriers, respectively. Against this background we decided to include these two factors as a measure of confidence in the final PPLQ version 2, while excluding the second factor. Moreover, there was a high degree of consensus of excluding the item CO5 from the first factor, due to similarity in content to item CO2. An inter-item correlation of .74 confirmed high redundancy between the two items. Running an EFA on the remaining six items from the two subscales, the two-factor structure could be confirmed (72.6% of the variance explained; Bartlett test p < .001; KMO criterion of .78). All factor loadings were over 0.5 (range between .719 and .882), with no cross loading detected.

**Domain: Physical Activity behavior**

Since PA behavior is represented by a formative measurement model, no EFA was conducted. Against this background the seven items of the International Physical Activity Questionnaire - Short Form (IPAQ-SF) were discussed only qualitatively with the experts in a joint meeting for a possibility of item reduction. There was a common consensus of excluding the item asking about time spent on sitting during the last 7 days. In their justifications, the experts referred to the essence of the PL concept i.e., sustained lifelong PA participation.^24^ They argued that, on the one hand, a person can have a physically active lifestyle even if they sit a lot. On the other hand, a person can also be physically inactive even if he or she does not sit much (e.g., by performing only very low-intensity activities such as standing). Therefore, the expert committee concluded that sedentary time might not be of primary relevance for operationalizing PL.

**Domain: Knowledge**

For the multiple-choice items (KN2 & KN3; see Table A.9), the numbers of correct answers were summed in each case prior to the EFA. Contrary to our assumption of the domain knowledge to be two-dimensional, a three-factor solution was revealed (see Table A.9), with an inadequate explained variance level of 45.8%. The Bartlett test was significant (p < .001), but the KMO criterion of .59 was insufficient. While an adequate factor loading pattern was found for the items assigned to the first and second factor, the two items (KN11 & KN12) on the third factor showed both loadings below .50 and cross loadings over .40. Against this background, these two items were excluded in this first step. An EFA on the remaining nine items revealed a two-factor solution (50.9% of the variance explained; Bartlett test p < .001; KMO criterion of .61), with an adequate factor loading pattern. Following a theory-driven approach (see Table A.1, domain knowledge), the two factors could clearly be interpreted as "how to move" (first factor; item KN1, KN5, KN6, KN7 & KN8) and "knowledge of benefits" (second factor; item KN2 & KN3). The factor loadings were in an adequate range between .544 and .837, with no substantial cross loadings observed. The Cronbach’s alpha coefficients was .67 for “how to move” and .61 for "knowledge of benefits", indicating borderline levels of sufficient internal consistency. We also calculated the item difficulty of each item. Apart from item KN5, which was found to be too easy (i.e., 93% answered it correct), the item difficulties for all items were in an adequate range between 20% and 80%.

In a second step, the results were presented to the seven experts. To identify further opportunities for an item reduction, they were asked to rate the remaining five items from the first factor either as “relevant (x)” or “non relevant (-)”. Besides, they were asked to justify their answers briefly (open-end question). The results from the expert rating are illustrated in Table A.10. All items reached an adequate level of relevance within the expert rating, with a CVI of 1.00 (seven out of seven) for item KN5, KN6 and KN8 and 0.86 (six out of seven) for the item KN1 and KN7. Based on the quantitative and qualitative results from the expert rating, a common consensus was reached to include all five items as a measure of the knowledge subscale “how to move” in the final PPLQ version 2. Based on the recommendation of four experts, we decided to increase the item difficulty of item KN5 by expanding the response scale, which also resulted in a slight rewording of the item to “Up to what age is muscle strength trainable” [40, 50, 60, 70, 80, 90, always]”. Moreover, since the subscale “knowledge of benefits” finally consisted of only two items (with a multiple-choice response format), we reached a consensus in the discussion with the expert to add the answer options “heart failure” to item KN3 to obtain a more sophisticated measure.

Rational for the item selection of the subscale “how to move” (summary of the expert’s statements):

Overall, all seven experts stated that the five items in their entirety provide a sophisticated measure of knowledge related to current PA guidelines for adults,^20,21^ with no content similarities noted between items. Due to a lack of evidence on how to assess PA knowledge,^25^ they argued that it is very difficult at this stage to classify individual items as "non relevant”. Further research might be needed to make a proper decision regarding the relevance of these items. Specifically, the experts stated that they rated KN5, KN6, KN7 and KN8 as relevant since these items showed good factor loadings and are concisely regarding the wording. In contrast, the majority of experts indicated that item KN1 had been at the bottom of the relevance ranking due to its length of the wording and low factor loading. However, they commented that they still considered it as relevant since it was the only item that assessed endurance-related PA knowledge. Additionally, item KN8 was rated as "non relevant" by one expert because its content may not be equally relevant for both genders. Moreover, even though item KN5 was considered very appropriate as a measure of PA knowledge, four experts recommended adjusting the wording and the answer scale of this item, since its item difficulty was inappropriate.

**Table A.9:** Factor matrix of the domain knowledge after varimax rotation

| Items | | Factorial loadings | | | |
| --- | --- | --- | --- | --- | --- |
|  | **Strength subscale** |  |  |  |  |
| ST1 | I am a physically strong person | .845 |  |  |  |
| ST2 | I would do well in a test of strength | .838 |  |  |  |
| ST3 | I have a lot of power in my body | .877 |  |  |  |
|  | **Endurance subscale** |  |  |  |  |
| EN1 | I can be physically active for a long period of time without getting tired |  | .778 |  |  |
| EN2 | I think I could run a long way without getting tired |  | .631 |  |  |
| EN3 | I am good at endurance activities e.g., distance run, aerobics, cycling, swim or cross-country ski |  | .890 |  |  |
|  | **Coordination subscale** |  |  |  |  |
| CO1 | I feel confident in performing difficult and rapid successive movements |  |  | .629 |  |
| CO2 | I find it easy to control the movements of my body |  |  | .780 |  |
| CO3 | I can coordinate my movements well |  |  | .825 |  |
| CO4 | In most physical activities, my movements are smooth and even |  |  | .795 |  |
| CO5 | I find my body can make steady movements easily |  |  | .787 |  |
|  | **Sport subscale** |  |  |  |  |
| SP1 | I'm good at most sports |  |  |  | .749 |
| SP2 | I have good sporting skills | .504 |  |  | .568 |
| SP3 | I'm good at sports games |  |  |  | .888 |

**Table A.2:** Factor matrix of four selected subscales from the PSDQ-S after Varimax rotation

|  | Items | Factor loadings | | |
| --- | --- | --- | --- | --- |
| KN1 | According to the Austrian PA guidelines, at least how many minutes per week should you perform endurance-oriented activities that involve a slight increase in breathing and pulse rate (e.g., brisk walking)? [30 min, 45 min, 60 min, 75 min, 90 min, 120 min, **150 min**, 180 min, 240 min] | .512 |  |  |
| KN2 | A physically inactive lifestyle increases the risk of suffering the following diseases [**breast cancer, dementia, hypertension**] |  | .811 |  |
| KN3 | PA can improve the course of the following diseases” **[sugar disease (diabetes mellitus type II) Parkinson's disease, joint wear (arthrosis)]** |  | .826 |  |
| KN4 | Three 10-minutes sessions of PA three times per week provide the same health benefits as a single 30-minutes-sessions” [**true**/false] |  | .423 | .437 |
| KN5 | Strength can only be trained up to the age of 60 [true, **false**] | .681 |  |  |
| KN6 | Strength training alone (without endurance training) does not result in any health benefits [true, **false**] | .706 |  |  |
| KN7 | Women need different strength exercises than men to build muscle [true, **false**] | .677 |  |  |
| KN8 | Strength training is not suitable for losing weight (body fat) [true, **false**] | .688 |  |  |
| KN9 | A person (80kg) consumes how many kilocalories per hour (approximately) through normal walking [50 kcal, 100 kcal, **300 kcal,** 500 kcal, 800 kcal, 1000 kcal] |  | .408 | .485 |

**Table A.10:** Expert ratings on the relevance of the item within the knowledge domain

**Table A.3:** Expert ratings on the relevance of the physical competence subscales

| Subscales | Experts | | | | | | | CVI |
| --- | --- | --- | --- | --- | --- | --- | --- | --- |
|  | 1 | 2 | 3 | 4 | 5 | 6 | 7 |  |
| KN1 | x | x | - | x | x | x | x | 0.86 |
| KN5 | x | x | x | x | x | x | x | 1.00 |
| KN6 | x | x | x | x | x | x | x | 1.00 |
| KN7 | x | x | x | - | x | x | x | 0.86 |
| KN8 | x | x | x | x | x | x | x | 1.00 |

**A3. Stage 3**

**A.3. Methods: Stage 3**

The aim of stage 3 was to improve content clarity and interpretation consistency of the remaining items from the PPLQ version 2, as important components of content validity. For this purpose, the cognitive interviewing method was used.^26^ This qualitative research technique involves individual interviews in which respondents verbalize their thought processes as they interact with the items to identify potential problems with wordings, response options, and overall questionnaire design. Adapting a theoretical sampling strategy,^27^ two rounds of four and three cognitive interviews were conducted, respectively. The analysis of the interviews from stage two indicated a saturation (i.e., no new information was provided within the interviews in round two). Participants were recruited through the authors' personal networks in March 2021 and were selected to achieve a high heterogeneity in terms of socioeconomic status (i.e., highest level of education) and PA history. Out of the seven participant, 71% (n = 5) were female. The mean was 54.71 ± 2.29 years and distribution of the participants' highest education level was as follows: 29% (n = 2) compulsory school; 29% (n = 2 apprenticeship, 29% (n = 2) college/A level and 13% (n = 1) university degree. All interviews were conducted in person, tape-recorded, transcribed verbatim, and subsequent summarized for each domain.

**A.3. Results: Stage 3**

Table A.11 presents the summarized results per domain from the seven cognitive interviews. All results were discussed within the expert committee from stage 2 to achieve consensus regarding possible changes. All changes made are described in the right column of Table A.11. Most notably, the three items measuring vigorous, moderate, and walking PA (days and hours/minutes per week) within the PA behavior domain were visually combined into one item each. The revision procedure in this stage resulted in a PPLQ version 3 of 31 items.

**Table A.11:** Results from the cognitive interviews in stage 3

**Table A.3:** Results from the cognitive interviews in stage 3

| **Domain** | **Interview comments (summary)** | **Actions done** |
| --- | --- | --- |
| Physical competence | 1. Regarding the item “*I am a physically strong person”*, it was difficult for the participants to interpret the term “strong”, since the term has many meaning within the Austrian linguistic culture (e.g., "having a lot of muscular strength" or "being overweight or adipose") (n = 3). 2. Regarding the item “*I have a lot of power in my body*” it was difficult for the participants to interpret the phrase “power in my body” (e.g., it was also associated with mental power) (n = 4). 3. Regarding the item “*I would do well in a test of strength*”, participants could not imagine anything about a strength test  (n = 2) or did not know which strength (mental or physical) was meant (n = 1). 4. Regarding the item “*I think I could run a long way without getting tired”,* it was difficult for the participants to interpret the terms “long way” (e.g., it was asked about the definition/kilometer count of a long way) (n = 3) | 1. Item was reworded to “*I have a lot of muscle power.*” 2. Item was replaced by an item adapted from the PAHCO questionnaire *”It is easy for me to lift heavy objects (e.g., full beverage crate)”.* 3. Item was reworded to “*I would do well in a test of muscle strength”* (the term “muscle” was added). 4. Item was reworded to *“I can run for at least 30 minutes without stopping”.* |
| Understanding | no comments given | n.a. |
| Motivation | no comments given | n.a. |
| Confidence  (i.e., self-efficacy) | no comments given | n.a. |

**Table A.11 (continued):** Results from the cognitive interviews in stage 3

**Table A.3 (continued):** Results from the cognitive interviews in stage 3

| **Domain** | **Interview comments (summary)** | **Actions done** |
| --- | --- | --- |
| PA behavior | 1. It was difficult for the participants to distinguish between moderate and vigorous intensity PA (n = 4). 2. The two questions about "days per week" and corresponding time (hours & minutes) spend on e.g., moderate intensity PA were not perceived as coherent (n = 2). 3. Regarding the number of days and time spent on PA in moderate intensity, participants included walking, even it was quoted not to include it (n = 2). | 1. Simple explanations for moderate and vigorous activities were added to the prefaced explanatory text. 2. The items regarding vigorous, moderate, and walking PA (days and hours/minutes per week), were visually combined to one item each. 3. The item(s) regarding walking PA (days and hours/minutes per week) was/were placed at the beginning of all items (i.e., before the items addressing moderate and vigorous PA). Moreover, for the vigorous and moderate PA items, it was specifically noted and highlighted not to include walking time. |
| Knowledge | 1. Regarding the item “*Austrian PA guidelines, at least how many minutes per week should you perform endurance-oriented activities that involve a slight increase in breathing and pulse rate (e.g., brisk walking)? [30 min, 45 min, 60 min, 75 min, 90 min, 120 min, 150 min, 180 min, 240 min]”,* participants converted the minutes given as answer options to hours while answering the item (n = 2). | 1. Hourly data was added to the response scale: *30 min (½ hour), 45 min (¾ hours), 60 min (1 hour), 75 min (1 ¼ hours), 90 min (1½ hours), 120 min (2 hours), 150 min (2½ hours), 180 min (3 hours), 240 min (4 hours).* |

**A4. Stage 4**

**A.4. Methods: Stage 4**

The aim of this stage was to ensure a language level of A2 for the revised questionnaire version from stage 3, which allows the PPLQ to be classified as an easy-to-understand questionnaire (i.e., 96% of the population should understand it).^28^ For this purpose, the questionnaire was subjected to an external comprehensibility check by *capito* (www.capito.eu), a certified organization for barrier-free and comprehensible information and communication. In a first step, the wording and overall design of the questionnaire were assessed against 90 formal- and content-related quality criteria, all of which must be met for the questionnaire to be assigned to language level of A2 (e.g., № 34: only participle II in the present perfect tense is permissible). In a second step, the modified questionnaire was check by three representatives from the target group to detect possible ambiguities and misunderstandings. All suggested changes were discussed by *capito* with the study authors to reach a consensus regarding their implementation.

**A.4. Results: Stage 4**

Table A.12 presents the results from the external comprehensibility check by *capito* as well as the subsequent implemented changes in final questionnaire version from stage 4. Overall, the modifications conducted within the four stages resulted in a PPLQ version with 31-item and 6 domains (see Table A.13).

**Table A.12:** Results from the external comprehensibility check in stage 4

**Table A.4 (continued):** Results from the external comprehensibility check in stage 4

|  | **Comments** | **Actions done** |
| --- | --- | --- |
| **General** | 1. Consider a revision of the introduction of the questionnaire since it is partly difficult to understand, especially for persons with low reading competence (i.e., persons with only an A2 language level or lower). 2. To minimize the risk of a left-side bias (primary effect), a decremental succession of the response scale is recommended for the domain physical competence, motivation, confidence (i.e., self-efficacy) and understanding, (i.e., “strongly agree” should be placed at the left end of the scale). Moreover, individuals with low reading competence are more familiar with the above-mentioned response scale format. | 1. The introduction of the questionnaire was revised according to the rules of " Easy Language” (e.g., sentences were shortened or divided into several sentences etc.). 2. The response scale for the domain physical competence, motivation, confidence (i.e., self-efficacy) and understanding has been reversed from 5 (= strongly agree) to 0 (= strongly disagree). |
| **Domains** |  |  |
| Physical competence | no comments given | n.a. |
| Understanding | 1. Consider shortening and positively rewording the text of all items to increase readability. | (i) All three items within this domain were shortened and positively reworded. |
| Motivation | 1. Consider a revision of the introductory sentence *“I intend to be regularly active in sports in the coming weeks and months,…”,* to ensure an easy-to-understand text level and a consistent wording regarding sport and PA in the questionnaire. | 1. The introductory sentence was reworded to “*I plan to be physically active on a regular basis in the coming weeks and months, …”. S*ince, in contrast to the international terminology, sport and PA are used synonymously in German-speaking countries, the term “sports” was replaced by a PA related term, to ensure a consistent wording within the questionnaire, which is also in line with PL definition of the International Physical Literacy Association (IPLA).^29^ |

|  | **Comments** | **Actions done** |
| --- | --- | --- |
| **Domains** |  |  |
| Confidence  (i.e., self-efficacy) | 1. Consider a revision of the introductory sentence *“I am sure that I will still be able to perform a planned sports activity even if...”* to ensure an easy-to-understand text level and a consistent wording regarding sport and PA in the questionnaire. | 1. The introductory sentence was reworded to “I still engage in planned physical activities even if...”.  *S*ince, in contrast to the international terminology, sport and PA are used synonymously in German-speaking countries, the term “sports” was replaced by a PA related term, to ensure a consistent wording within the questionnaire, which is also in line with PL definition of the International Physical Literacy Association (IPLA).^29^ |
| PA behavior | 1. Consider a revision of the prefaced explanatory text. | 1. The prefaced explanatory text was revised according to the rules of “Easy Language” (e.g., sentences were shortened or divided into several sentences, additional paragraphs breaks were inserted etc.). |
| Knowledge | 1. Consider a revision of the prefaced explanatory text. 2. Consider a revision of the *item “According to the Austrian PA guidelines, at least how many minutes per week should you perform endurance-oriented activities that involve a slight increase in breathing and pulse rate (e.g., brisk walking)?”* - the term “endurance-oriented activities” is difficult to understand. 3. Consider a positively rewording of the item *“Strength training alone (without endurance training) does not result in any health benefits”* to ensure an easy-to-understand text level. 4. Consider a positively rewording of the item *“Strength training is not suitable for losing weight (body fat)”*. | 1. The prefaced explanatory text was revised according to the rules of “Easy Language” (e.g., sentences were shortened or divided into several sentences). 2. The item was reworded to *“According to the Austrian PA guidelines, at least how many minutes per week should you perform activities that involve a slight increase in breathing and pulse rate e.g., brisk walking)?”.* 3. The item was reworded to *“Pure strength training (without endurance training) also has health benefits”* 4. The item was reworded to *“Strength training is suitable for losing weight (body fat)”*. |

**Table A.12 (continued):** Results from the external comprehensibility check in stage 4

**Table A.4 (continued):** Results from the external comprehensibility check in stage 4

**Table A.12 (continued):** Results from the external comprehensibility check in stage 4

**Table A.4 (continued):** Results from the external comprehensibility check in stage 4

**Table A.13:** 31-item version of the PPLQ resulted from stage 4 (PPLQ version 4)

**Table A.5:** 31-item version of the PPLQ resulted from stage 4

|  | **Measurement** |
| --- | --- |
| **General** | - The revises questionnaire at the end of stage 1 (PPLQ version 1) included in the domain physical competence, motivation, confidence (i.e., self-efficacy) and understanding an uniform Likert scale ranging from 1 (= strongly disagree) to 6 (= strongly agree). Within stage 4 the response scale has been reversed from 5 (= strongly agree) to 0 (= strongly disagree). - The introduction of the questionnaire was revised according to the rules of “Easy Language” (e.g., sentences were shortened or divided into several sentences) ^Stage 4^ |
| **Domains** |  |
| Physical competence [PCO]  2 subfactors:   - strength (PCO_ST; 3 items) - endurance (PCO_EN; 3 items) | Physical Self-Description Questionnaire - Short Version  (PSDQ-S),^3,4^ endurance (3 items) and strength subscales (3 items)  **General modification:**  The revised questionnaire at the end of stage 1 (PPLQ version 1) included four subscales from the PSDQ-S: endurance, strength, coordination, and sport competence. Within stage 2, the subscales coordination and sport competence were excluded.  **Introductory sentence modification (prefaced stem to all items):** n/a  **Item modifications (strength subscale):**   1. *“I am a physically strong person”* - item was reworded to *“I have a lot of muscle power”* ^Stage 3^ 2. *“I have a lot of power in my body”* – item was replaced by an item adapted from the PAHCO^30^ *”It is easy for me to lift heavy objects (e.g., full beverage crate)”* ^Stage 3^ 3. “*I would do well in a test of strength” -* item was reworded to *“I would do well in a test of muscle strength”* (only the term “muscle” was added) ^Stage 3^   **Item modifications (endurance subscale):**   1. *“I think I could run a long way without getting tired”* - item was reworded to *“I can run for at least 30 minutes without stopping”* ^Stage 3^ 2. “*I can be physically active for a long period of time without getting tired”* (no modification) 3. *“I am good at endurance activities e.g., distance run, aerobics, cycling, swim or cross-country ski” -* brackets were added: *“I am good at endurance activities (e.g., distance run, aerobics, cycling, swim or cross-country ski)”* |
| Understanding [UND]  1 factor (3 items) | Two out of three items were adapted from Sum et al. (2018)^6^ and Brooks et al. (2018),^7^ respectively. One item was self-constructed.  **General modification:**  The revised questionnaire at the end of stage 1 (PPLQ version 1) included 7 items (see Table A.1). Within stage 2, only 3 items were selected.  **Introductory sentence modification (prefaced stem to all items):** n/a  **Item modifications:**   1. *“I think engaging in PA regularly is a waste of time”* ^(adapted from Brooks et al., 2016)^ - item was positively reworded to *“I see a purpose in engaging in PA regularly”* ^Stage 4^ 2. *“I feel less appreciation for other people engaging in regular PA”* ^(adapted from Sum et al., 2018)^ - item was shortened and positively reworded to *“I feel a lot of appreciation for people engaging in regular PA”* ^Stage 4^ 3. *“I consider initiatives in companies or organizations to increase PA (e.g., company walking day) to be superfluous”* ^(self-constructed)^ - item was shortened and positively reworded to: *“I think initiatives in companies to increase PA (e.g., company walking day) make sense”* ^Stage 4^ |

|  | **Measurement** |
| --- | --- |
| Motivation [MOT]  2 subfactors:   - intrinsic motivation  (MOT_IN; 3 items) - identified motivation  (MOT_ID; 3 items) | Sport-and Exercise-Related Self-Concordance-Scale (SSK-Scale),^8^ intrinsic (3 items) and identified motivation subscales (3 items)  **General modification:**  The revised questionnaire at the end of stage 1 (PPLQ version 1) included four subscales from the SSK-Scale: intrinsic, identified, introjected and extrinsic motivation. Within stage 2, the subscales introjected and extrinsic motivation were excluded.  **Introductory sentence modification (prefaced stem to all items):**  *“I intend to be regularly active in sports in the coming weeks and months…”* - reworded to *“I plan to be physically active on a regular basis in the coming weeks and months…”* ^Stage 4^  **Item modifications (intrinsic motivation):**   1. *“… because I simply enjoy it”* (no modification) 2. *“…because sporting activity is simply part of my life”*- item was reworded to *“... because PA is simply part of my life”* (“sporting activity” was replaced by “PA”) ^Stage 4^ 3. *“…because it gives me experiences that I wouldn't want to miss”* (no modification)   **Item modifications (identified motivation):**   1. *“…because the positive consequences are simply worth the effort”* (no modification) 2. *“…because it is good for me”* (no modification) 3. *“…because I have good reasons for it”* (no modification) |
| Confidence (i.e., self-efficacy) [CON]  2 subfactors:   - internal barriers  (CON_IB; 3 items) - external barriers (CON_EB; 3 items) | Self-efficacy for sports activity scale (SSA scale)^9^  **General modification:**  The revised questionnaire at the end of stage 1 (PPLQ version 1) included all 12 items of the SSA scale.  Within stage 2, only 6 items were selected.  **Introductory sentence modification (prefaced stem to all items):**  “*I am sure that I will still be able to perform a planned sports activity even if..*.” – reworded to “*I still engage in planned physical activities even if...*” ^Stage 4^  **Item modifications (internal barriers):**   1. *“... I am tired”* (no modification) 2. *“… I feel depressed”* (no modification) 3. *“…I am annoyed about something”* (no modification)   **Item modifications (external barriers):**   1. *“... I can't find anyone to do sports with me”* (no modification) 2. *“... the weather is bad”* (no modification) 3. *“… an interesting TV program is running”* (no modification) |
| PA Behavior  (PAB; 3 items) | International Physical Activity Questionnaire - Short Form (IPAQ-SF),^10^ without the item for “sedentary behavior”  **General modification:**  The revised questionnaire at the end of stage 1 (PPLQ version 1) included all seven items for dem IPAQ-SF. Within stage 2, the item for “sedentary behavior” was excluded. Moreover, within stage 4 the prefaced explanatory text to the questionnaire was modified to a A2 language level and extended (e.g., simple explanations for moderate and vigorous activities were added).  **Item modifications:**   - The items regarding vigorous, moderate, and walking physical activities (days and hours/minutes per week), were visually combined to one item each (for this reason, we currently count these only as 1 item each) ^Stage 3^ - The item(s) regarding walking physical activities (days and hours/minutes per week) was/were placed at the beginning of all items (i.e., before the items addressing moderate and vigorous PA ). Moreover, for the vigorous and moderate PA items, it was specifically noted and highlighted not to include walking time ^Stage 3^ |

**Table A.13 (continued):** 31-item version of the PPLQ resulted from stage 4 (PPLQ version 4)

**Table A.5 (continued):** 31-item version of the PPLQ resulted from stage 4

|  | **Measurement** |
| --- | --- |
| Knowledge [KNO]  2 subfactors:   - how to move  (KNO_HM; 5 items) - knowledge of the benefits (KNO_KB; 2 items) | Three out of seven items were adapted from Morrow et al. (2004),^11^ Hui and Morrow (2001)^12^ and Rowland et al. (1994).^13^ Four items were self-constructed.  **General modification:**  The revised questionnaire at the end of stage 1 (PPLQ version 1) included 9 items regarding the domain knowledge (see Table A.1). Within stage 2, only 7 items were selected. Moreover, within stage 4 the prefaced explanatory text to the questionnaire was modified to a A2 language level.    **Item modifications (how to move;** correct answers in bold**):**   1. *“Strength can only be trained up to the age of 60” [true,* ***false****]* ^(self-constructed)^ - item was reworded and the response scale was expanded to *“Up to what age is muscle strength trainable” [40, 50, 60, 70, 80, 90,* ***always****]”* ^Stage 2^ 2. *“According to the Austrian PA guidelines, at least how many minutes per week should you perform endurance-oriented activities that involve a slight increase in breathing and pulse rate (e.g., brisk walking)?” [30 min, 45 min, 60 min, 75 min, 90 min, 120 min,* ***150 min****, 180 min, 240 min]* ^(adapted from Morrow et al., 2014)^ - hourly data was added to the response scale and the item was reworded to *“According to the Austrian PA guidelines, at least how many minutes per week should you perform activities that involve a slight increase in breathing and pulse rate e.g., brisk walking)? [30 min (½ hour), 45 min (¾ hours), 60 min (1 hour), 75 min (1 ¼ hours), 90 min (1½ hours), 120 min (2 hours),* ***150 min (2½ hours)****, 180 min (3 hours), 240 min (4 hours)]* ^Stage 3 & 4.^ 3. *“Strength training alone (without endurance training) does not result in any health benefits” [true,* ***false****]* ^(self-constructed)^ *-* item was positively reworded to “Pure strength training (without endurance training) also has health benefits *[****true****, false]”* ^Stage 4^ 4. *“Women need different strength exercises than men to build muscle” [true,* ***false****]* ^(self-constructed)^ (no modification) 5. *“Strength training is not suitable for losing weight (body fat)” [true,* ***false****]* ^(self-constructed)^ *-* item was positively reworded to “*“Strength training is suitable for losing weight (body fat)” [****true****, false]”* ^Stage 4^   **Item modifications (knowledge of the benefits;** correct answers in bold**):**   1. *“A physically inactive lifestyle increases the risk of suffering the following diseases [****breast cancer, dementia, hypertension****]”* ^(adapted from Hui and Morrow, 2001)^ (no modification) 2. *“PA can improve the course of the following diseases [****sugar disease (diabetes mellitus type II) Parkinson's disease, joint wear (arthrosis)****]”* ^adapted from Roland et al., 1994)^ *-* extension of the answer options with **“heart failure”** also presenting a correct answer) ^Stage 2^ |

**Table A.13 (continued):** 31-item version of the PPLQ resulted from stage 4 (PPLQ version 4)

**Table A.5 (continued):** 31-item version of the PPLQ resulted from stage 4

**A5. Stage 5**

Figure A.1 presents the higher-order model of the PPLQ version 4 tested in stage 5.

**
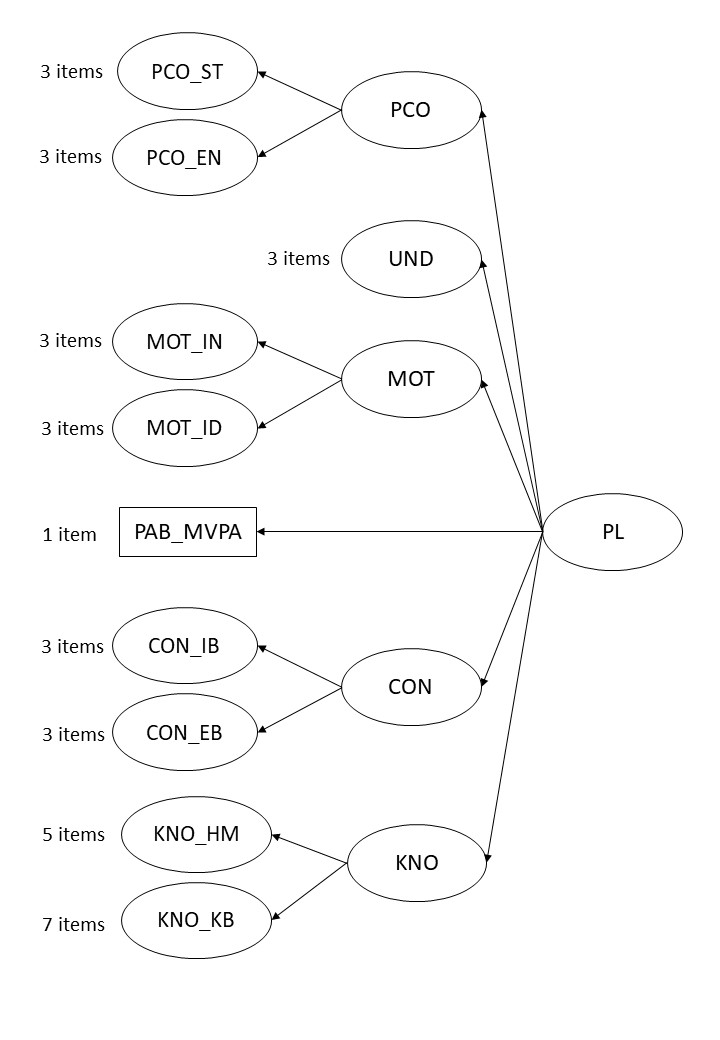
**

**Figure A.1:** Higher-order model of the 31-item-version of the PPLQ version 4. For better visualization, only the structural model and the respective number of manifest variables are presented. The domain PA behavior enters the model as a formative construct with a single indicator (see section 2.5. in the manuscript for detailed information). PL: Physical Literacy; PCO: domain physical competence; PCO_ST: sub-domain strength; PCO_EN: sub-domain endurance; UND: domain understanding; MOT: domain motivation; MOT_IN: sub-domain intrinsic motivation; MOT_ID: sub-domain identified motivation; CON: domain confidence (i.e., self-efficacy); CON_IB: sub-domain internal barriers; CON_EB: sub-domain external barriers; PAB: domain Physical activity behavior; KNO: domain knowledge; KNO_HM: sub-domain how to move; KNO_KB: sub-domain knowledge of the benefits.

**References of Appendix A**

1. Holler P, Jaunig J, Moser O, et al. Primary Care and physical literacy: A Non-Randomized Controlled Pilot Study to Combat the High Prevalence of Physically Inactive Adults in Austria. *Int J Environ Res Public Health*. 2021;18(16):8593. doi:10.3390/ijerph18168593

2. Holler P, Jaunig J, Amort FM, et al. Holistic physical exercise training improves PHYSICAL LITERACY among physically inactive adults: a pilot intervention study. *BMC Public Health*. 2019;19(1):393. doi:10.1186/s12889-019-6719-z

3. Marsh HW, Martin AJ, Jackson S. Introducing a short version of the physical self description questionnaire: new strategies, short-form evaluative criteria, and apphysical literacyications of factor analyses. *J Sport Exerc Psychol*. 2010;32(4):438-482. doi:10.1123/jsep.32.4.438

4. Braun A, Titus M, Alfermann D, Michel S. Überprüfung der Reliabilität und Validität der Kurzform des Physical Self-Description Questionnaire (PSDQ-S) in den Altersgruppen des frühen und späten Erwachsenenalters. *Z Für Sportpsychol*. 2018;25(3):115-127.

5. Fitgerald JT, Singleton SP, Neale AV, Prasad AS, Hess JW. Activity Levels, Fitness Status, Exercise Knowledge, and Exercise Beliefs among Healthy, Older African American and White Women. *J Aging Health*. 1994;6(3):296-313. doi:10.1177/089826439400600302

6. Sum RKW, Cheng CF, Wallhead T, Kuo CC, Wang FJ, Choi SM. Perceived Physical Literacy instrument for adolescents: A further validation of PPLI. *J Exerc Sci Fit*. 2018;16(1):26-31. doi:10.1016/j.jesf.2018.03.002

7. Brooks JM, Kaya C, Chan F, et al. Validation of the Behavioural Regulation in Exercise Questionnaire-2 for adults with chronic musculoskeletal pain. *Int J Ther Rehabil*. 2018;25(8):395-404. doi:10.12968/ijtr.2018.25.8.395

8. Seelig H, Fuchs R. Messung der sport- und bewegungsbezogenen Selbstkonkordanz. *Z Für Sportpsychol*. 2006;13(4):121-139. doi:10.1026/1612-5010.13.4.121

9. Fuchs R, Schwarzer R. Selbstwirksamkeit zur sportlichen Aktivitaet: Reliabilitaet und Validitaet eines neuen Messinstruments. *Z Für Differ Diagn Psychol*. 1994;15(3):141-154.

10. Craig CL, Marshall AL, Sjöström M, et al. International Physical Activity questionnaire: 12-country reliability and validity. *Med Sci Sports Exerc*. 2003;35(8):1381-1395. doi:10.1249/01.MSS.0000078924.61453.FB

11. Morrow JR, Krzewinski-Malone JA, Jackson AW, Bungum TJ, FitzGerald SJ. American adults’ knowledge of exercise recommendations. *Res Q Exerc Sport*. 2004;75(3):231-237. doi:10.1080/02701367.2004.10609156

12. Hui S, Morrow J. Level of Participation and Knowledge of PA in Hong Kong Chinese Adults and Their Association with Age. *J Aging Phys Act*. 2001;9(4):372-385. doi:10.1123/japa.9.4.372

13. Rowland L, Dickinson EJ, Newman P, Ford D, Ebrahim S. Look After Your Heart programme: impact on health status, exercise knowledge, attitudes, and behaviour of retired women in England. *J Epidemiol Community Health*. 1994;48(2):123-128.

14. Edwards LC, Bryant AS, Keegan RJ, Morgan K, Jones AM. Definitions, Foundations and Associations of Physical Literacy: A Systematic Review. *Sports Med Auckl Nz*. 2017;47(1):113-126. doi:10.1007/s40279-016-0560-7

15. Widaman KF, Little TD, Preacher KJ, Sawalani GM. On creating and using short forms of scales in secondary research. In: Trzesniewski KH, Donnellan MB, Lucas RE, eds. *Secondary Data Analysis: An Introduction for Psychologists*. American Psychological Association; 2011:39-62.

16. Zamanzadeh V, Ghahramanian A, Rassouli M, Abbaszadeh A, Alavi-Majd H, Nikanfar AR. Design and Implementation Content Validity Study: Development of an instrument for measuring Patient-Centered Communication. *J Caring Sci*. 2015;4(2):165-178. doi:10.15171/jcs.2015.017

17. Lynn MR. Determination and quantification of content validity. *Nurs Res*. 1986;35(6):382-385.

18. Jones GR, Stathokostas L, Young BW, et al. Development of a Physical Literacy model for older adults – a consensus process by the collaborative working group on PL for older Canadians. *BMC Geriatr*. 2018;18(1):13. doi:10.1186/s12877-017-0687-x

19. Peterson MJ, Giuliani C, Morey MC, et al. Physical activity as a preventative factor for frailty: the health, aging, and body composition study. *J Gerontol A Biol Sci Med Sci*. 2009;64(1):61-68. doi:10.1093/gerona/gln001

20. World Health Organization. WHO guidelines on Physical activity and sedentary behavior. Accessed November 21, 2022. https://www.who.int/publications-detail-redirect/9789240015128

21. *Österreichische Bewegungsempfehlungen*. https://fgoe.org/sites/fgoe.org/files/2020-06/WB17_bewegungsempfehlungen_bfrei.pdf

22. PL. Consensus Statement. Physical Literacy. Published 2022. Accessed November 21, 2022. https://physicalliteracy.ca/physical-literacy/consensus-statement/

23. Deci EL, Ryan RM. Self-determination theory: A macrotheory of human motivation, development, and health. *Can Psychol Psychol Can*. 2008;49(3):182-185. doi:10.1037/a0012801

24. Francis CE, Longmuir PE, Boyer C, et al. The Canadian Assessment of Physical Literacy: Development of a Model of Children’s Capacity for a Healthy, Active Lifestyle Through a Delphi Process. *J Phys Act Health*. 2016;13(2):214-222. doi:10.1123/jpah.2014-0597

25. Ryom K, Hargaard AS, Melby PS, et al. Self-reported measurements of Physical Literacy in adults: a scoping review. *BMJ Open*. 2022;12(9):e058351. doi:10.1136/bmjopen-2021-058351

26. Balza JS, Cusatis R, McDonnell SM, Basir MA, Flynn KE. Effective questionnaire design: How to use cognitive interviews to refine questionnaire items. *J Neonatal-Perinat Med*. 2022;15(2):345-349. doi:10.3233/NPM-210848

27. Strauss AL, Corbin JM. *Basics of Qualitative Research: Techniques and Procedures for Developing Grounded Theory*. 2nd ed. Sage Publications; 1998.

28. capito. Easy-to-understand language and barrier-free informationeichte Sprache - Begriffe, Regeln und Beispiele. capito - Leichte Sprache. Accessed January 27, 2023. https://www.capito.eu/leichte-sprache/

29. International PL Association. International Physical Literacy Association. IPLA. Published 2022. Accessed November 20, 2022. https://www.physical-literacy.org.uk/

30. Carl J, Sudeck G, Geidl W, Schultz K, Pfeifer K. Competencies for a Healthy Physically Active Lifestyle—Validation of an Integrative Model. *Res Q Exerc Sport*. 2021;92(3):514-528. doi:10.1080/02701367.2020.1752885
