# Supplementary material for: Development of the Perceived Physical Literacy Questionnaire (PPLQ) for the adult population
Source: J Exerc Sci Fit. 2023 Oct 5;21(4):424–33. doi: 10.1016/j.jesf.2023.09.003 (PMC10661355; doi:10.1016/j.jesf.2023.09.003)
Supplement: Multimedia component 2 [file mmc2.docx]

**Appendix B**

**Fragebogen zur Bewegungskompetenz (Physical Literacy)**

Sehr geehrte Teilnehmerin, sehr geehrter Teilnehmer!

Vielen Dank, dass Sie diesen Fragebogen ausfüllen.

**Bitte beantworten Sie alle Fragen vollständig.** Wenn Sie sich bei einer Frage unsicher sind, wählen Sie bitte die Antwort, die Ihrer Meinung nach am besten auf Sie zutrifft.

| **Angaben zu Ihrer Person** | | | |
| --- | --- | --- | --- |
| Geschlecht | männlich O | weiblich O | divers O |
| Wie alt sind Sie? | ___________________ Jahre | | |
| Wie groß sind Sie? | ___________________ cm | | |
| Gewicht | ___________________ kg | | |
| Was ist Ihre höchste erfolgreich abgeschlossene Schulbildung? | O Pflichtschule  O Lehrabschluss (Berufsschule)  O Berufsbildende mittlere Schule  O Höhere Schule mit Matura (z.B. AHS, BHS, Kolleg)  O Hochschulverwandte Lehranstalt (z.B. Akademie)  O Universität, Fachhochschule | | |

In diesem Teil geht es darum, wie Sie sich selbst beschreiben. Bitte lesen Sie jede Aussage sorgfältig durch. Entscheiden Sie dann, wie sehr die Aussage auf Sie zutrifft.

Bitte entscheiden Sie sich schnell für eine Antwort und kreuzen Sie diese an.

**Bitte beantworten Sie alle Aussagen**. Es gibt keine falschen Antworten.

|  | **trifft**  **sehr**  **zu** | **trifft**  **zu** | **trifft**  **etwas**  **zu** | **trifft**  **eher nicht**  **zu** | **trifft**  **nicht zu** | **trifft gar nicht zu** |
| --- | --- | --- | --- | --- | --- | --- |
| Ich habe viel Muskelkraft. | ○ | ○ | ○ | ○ | ○ | ○ |
| Ich kann mindestens 30 Minuten laufen ohne anzuhalten. | ○ | ○ | ○ | ○ | ○ | ○ |
| Es fällt mir leicht, schwere Gegenstände (z.B. volle Getränkekiste) anzuheben. | ○ | ○ | ○ | ○ | ○ | ○ |
| In einem Test, der meine Muskelkraft misst, wäre ich gut. | ○ | ○ | ○ | ○ | ○ | ○ |
| Ich kann eine lange Zeit körperlich aktiv sein ohne müde zu werden. | ○ | ○ | ○ | ○ | ○ | ○ |
| Ich bin gut in Ausdauersportarten (z.B. Langstreckenlauf, Aerobic, Radfahren, Schwimmen oder Skilanglauf). | ○ | ○ | ○ | ○ | ○ | ○ |

|  | **trifft**  **sehr**  **zu** | **trifft**  **zu** | **trifft**  **etwas**  **zu** | **trifft**  **eher nicht**  **zu** | **trifft**  **nicht zu** | **trifft gar nicht zu** |
| --- | --- | --- | --- | --- | --- | --- |
| Ich sehe einen Sinn darin, sich regelmäßig körperlich zu betätigen. | ○ | ○ | ○ | ○ | ○ | ○ |
| Ich empfinde viel Anerkennung für Personen, die sich regelmäßig körperlich betätigen. | ○ | ○ | ○ | ○ | ○ | ○ |
| Ich halte Initiativen in Firmen zur Steigerung der körperlichen Bewegung (z.B. Betriebswandertag) für sinnvoll. | ○ | ○ | ○ | ○ | ○ | ○ |

| **Ich habe vor, in den nächsten Wochen und Monaten**  **regelmäßig körperlich aktiv zu sein, ...** | | | | | | |
| --- | --- | --- | --- | --- | --- | --- |
|  | **trifft**  **sehr**  **zu** | **trifft**  **zu** | **trifft**  **etwas**  **zu** | **trifft**  **eher nicht**  **zu** | **trifft**  **nicht zu** | **trifft gar nicht zu** |
| ... weil es mir einfach Spaß macht. | ○ | ○ | ○ | ○ | ○ | ○ |
| ... weil die positiven Folgen einfach die Mühe wert sind. | ○ | ○ | ○ | ○ | ○ | ○ |
| ... weil körperliche Aktivität einfach zu meinem Leben dazugehört. | ○ | ○ | ○ | ○ | ○ | ○ |
| ... weil es gut für mich ist. | ○ | ○ | ○ | ○ | ○ | ○ |
| ... weil ich dabei Erfahrungen mache, die ich nicht missen möchte. | ○ | ○ | ○ | ○ | ○ | ○ |
| ... weil ich gute Gründe dafür habe. | ○ | ○ | ○ | ○ | ○ | ○ |

| **Ich übe geplante körperliche Aktivitäten auch dann noch aus, wenn …** | | | | | | |
| --- | --- | --- | --- | --- | --- | --- |
|  | **trifft**  **sehr**  **zu** | **trifft**  **zu** | **trifft**  **etwas**  **zu** | **trifft**  **eher nicht**  **zu** | **trifft**  **nicht zu** | **trifft gar nicht zu** |
| … ich müde bin. | ○ | ○ | ○ | ○ | ○ | ○ |
| … ich mich niedergeschlagen fühle. | ○ | ○ | ○ | ○ | ○ | ○ |
| … ich mich über etwas ärgere. | ○ | ○ | ○ | ○ | ○ | ○ |
| … ich niemanden finde, der mit mir Sport treibt. | ○ | ○ | ○ | ○ | ○ | ○ |
| … schlechtes Wetter ist. | ○ | ○ | ○ | ○ | ○ | ○ |
| … ein interessantes Fernsehprogramm läuft. | ○ | ○ | ○ | ○ | ○ | ○ |

In diesem Teil geht es darum, wie viel Zeit Sie sich in **den letzten 7 Tagen** körperlich betätigt haben. Wenn Sie im Moment **krank oder verletzt** sind, beziehen Sie sich bitte auf die 7 Tage vor der Krankheit oder Verletzung.

Bitte berücksichtigen Sie alle körperlichen Aktivitäten. Zum Beispiel

- im Rahmen Ihrer Arbeit,
- in Haus und Garten,
- Bewegung von einem Ort zum anderen,
- Erholung,
- Leibesübungen und Sport

Denken Sie an all Ihre Aktivitäten mit anstrengender und mittlerer Intensität in den **vergangenen 7 Tagen**.

**Anstrengende Aktivitäten** bezeichnen Aktivitäten, die starke körperliche Anstrengungen erfordern und bei denen Sie deutlich stärker atmen als normal. Beim Ausüben dieser Aktivitäten ist kein durchgehendes Gespräch mehr möglich.

**Mittlere Aktivitäten** bezeichnen Aktivitäten mit körperlicher Anstrengung bei denen Sie ein wenig stärker atmen als normal. Beim Ausüben dieser Aktivitäten können Sie noch ein durchgehendes Gespräch führen, aber nicht mehr singen.

**Frage 1:**

An wie vielen der vergangenen 7 Tage sind Sie **mindestens 10 Minuten** ohne Unterbrechung **zu Fuß** gegangen? Dazu gehören Gehstrecken daheim oder in der Arbeit, gehen um von einem Ort zu einem anderen zu gelangen, sowie alles andere Gehen zur Erholung, Bewegung oder Freizeit.

_______ **Tage pro Woche**  **○ Keine Wege zu Fuß**

Wie viel Zeit haben Sie für gewöhnlich an **einem** dieser Tage mit **Gehen** verbracht?

**_______ Minuten pro Tag**

**Frage 2:**

Denken sie nur an die körperlichen Aktivitäten die Sie für **mindestens 10 Minuten** ohne Unterbrechung verrichtet haben. An wie vielen der **vergangenen 7 Tage** haben Sie **anstrengende** körperliche Aktivitäten wie Aerobic, Laufen, schnelles Fahrradfahren oder schnelles Schwimmen verrichtet?

_______ **Tage pro Woche**  **○ Keine anstrengende Aktivität**

Wie viel Zeit haben Sie für gewöhnlich an **einem** dieser Tage mit **anstrengenden körperlicher Aktivität** verbracht?

**_______ Minuten pro Tag**

**Frage 3:**

Denken Sie erneut nur an die körperlichen Aktivitäten die Sie für **mindestens 10 Minuten** ohne Unterbrechung verrichtet haben. An wie vielen der **vergangenen 7 Tage** haben sie **mittlere** körperliche Aktivitäten, wie das Tragen leichter Lasten, Fahrradfahren bei gewöhnlicher Geschwindigkeit oder Schwimmen bei gewöhnlicher Geschwindigkeit verrichtet?

**Achtung: Hierzu zählt nicht zu Fuß gehen!**

_______ **Tage pro Woche**  **○ Keine mittlere Aktivität**

Wie viel Zeit haben Sie für gewöhnlich an **einem** dieser Tage mit **mittlerer körperlicher Aktivität** verbracht?

**_______ Minuten pro Tag**

Beantworten Sie die folgenden Fragen, **ohne** Hilfsmittel wie Google oder Ähnliches zu verwenden. Bitte antworten Sie zügig und entscheiden Sie sich umgehend für eine Antwort.

1. Bis zu welchem Lebensjahr ist die Muskelkraft trainierbar?

**○ 40 ○ 50 ○ 60 ○ 70 ○ 80 ○ 90 ○ immer**

1. Wie viele Minuten pro Woche sollten Sie Aktivitäten laut den Österreichischen Bewegungsempfehlungen mindestens durchführen, bei denen Atmung und Puls leicht zunehmen (z.B. flottes Gehen)?

**○ 30 min** (½ Stunde) **○ 45 min** (¾ Stunden) **○ 60 min** (1 Stunde)

**○ 75 min** (1 ¼ Stunden) **○ 90 min** (1½ Stunden) **○ 120 min** (2 Stunden)

**○ 150 min** (2½ Stunden) **○ 180 min** (3 Stunden) **○ 240 min** (4 Stunden)

1. Auch durch reines Krafttraining (ohne Ausdauertraining) erfährt man einen gesundheitlichen Nutzen.

**○ Trifft zu ○ Trifft nicht zu**

1. Ein körperlich **in**aktiver Lebensstil erhöht das Risiko folgende Krankheiten zu erleiden:

**○ Brustkrebs ○ Demenz ○ Bluthochdruck**

1. Frauen brauchen beim Krafttraining andere Übungen als Männer, um Muskeln aufzubauen.

**○ Trifft zu ○ Trifft nicht zu**

1. Körperliche Bewegung kann den Verlauf folgender Krankheiten verbessern:

**○ Zuckerkrankheit (Diabetes Typ II) ○ Parkinson**

**○ Gelenksabnützung (Arthrose) ○ Herzschwäche**

1. Krafttraining ist zum Abnehmen (von Körperfett) geeignet

**○ Trifft zu ○ Trifft nicht zu**

**Ende des Fragebogens. Vielen Dank für Ihre Teilnahme!**
