# Supplementary material for: Development of the Perceived Physical Literacy Questionnaire (PPLQ) for the adult population
Source: J Exerc Sci Fit. 2023 Oct 5;21(4):424–33. doi: 10.1016/j.jesf.2023.09.003 (PMC10661355; doi:10.1016/j.jesf.2023.09.003)
Supplement: Multimedia component 3 [file mmc3.docx]

**Appendix C**

| **Domains scores** |  |
| --- | --- |
| Physical competence | **Coding:** 5 (= strongly agree) to 0 (= strongly disagree)  **Score calculation:**   1. $\mathrm{PCO}_{\mathrm{mean}}=\frac{\text{PCO\_ST1 + PCO \_EN1 + PCO\_ST2 + PCO \_ST3 +} \text{PCO \_EN2 + PCO \_EN3}}{6}$ 2. $\mathrm{PCO}_{\mathrm{Score}}=\frac{\mathrm{PCO}_{\mathrm{mean}}}{5}\times100$ |
| Motivation | **Coding:** 5 (= strongly agree) to 0 (= strongly disagree)  **Score calculation:**   1. $\mathrm{MOT}_{\mathrm{mean}}=\frac{\text{(MOT\_IN1 × 2) + MOT \_ID1 + (MOT\_IN2 × 2) + MOT \_ID2 +(MOT\_IN3 × 2) + MOT\_ID3}}{6}$ 2. $\mathrm{MOT}_{\mathrm{Score}}=\frac{\mathrm{MOT}_{\mathrm{mean}}}{7,5}\times100$ |
| Confidence  (self-efficacy) | **Coding:** 5 (= strongly agree) to 0 (= strongly disagree)  **Score calculation:**   1. $\mathrm{CON}_{\mathrm{mean}}=\frac{\text{CON\_IB1 + CON\_IB2 + CON\_IB3 + CON\_EB1 +} \text{CON\_EB2 + CON\_EB3}}{6}$ 2. $\mathrm{CON}_{\mathrm{Score}}=\frac{\mathrm{CON}_{\mathrm{mean}}}{5}\times100$ |
| Understanding | **Coding:** 5 (= strongly agree) to 0 (= strongly disagree)  **Score calculation:**   1. $\mathrm{UND}_{\mathrm{mean}}=\frac{\text{UND1 + UND2 + UND3}}{3}$ 2. $\mathrm{UND}_{\mathrm{Score}}=\frac{\mathrm{UND}_{\mathrm{mean}}}{5}\times100$ |
| Physical activity behavior | **Score calculation:**   1. $\mathrm{PAB}_{\mathrm{raw}}\text{= (PAB2a × PAB2b × 2) + (PAB3a × PAB3b) }$ 2. Truncation: Values exceeding 300 minutes are truncated to be equal to 300 minutes 3. $\mathrm{PAB}_{\mathrm{Score}}=\left( -\frac{4}{5625}\times\left( \mathrm{PAB}_{\mathrm{raw}} \right)^{2} \right)+\left( \frac{41}{75}\times\mathrm{PAB}_{\mathrm{raw}} \right)$ |
| Knowledge | **Coding:** all correct answers are coded with 1 (= 12 correct answers in total within the 7 items; incorrect answers are coded with 0; note that multiple answers are possible for the items KNO_KB1 and KNO_KB2, with each answer scored separately).  **Score calculation:**   1. $\mathrm{KNO}_{\mathrm{SUM}}\text{= }\text{KNO\_HM1 + KNO\_HM2 + KNO\_HM3 + KNO\_KB1 + KNO\_HM4 + KNO\_KB2 + KNO\_HM5}\text{ }$ 2. $\mathrm{KNO}_{\mathrm{Score}}=\frac{\mathrm{KNO}_{\mathrm{SUM}}}{12}\times100$ |
| **Total Physical Literacy Score** | **Score calculation:**  $\mathrm{PL}_{\mathrm{SCORE}}=\frac{\mathrm{PCO}_{\mathrm{Score}}\text{ + }\mathrm{MOT}_{\mathrm{Score}}\text{ + }\mathrm{CON}_{\mathrm{Score}}+ \mathrm{UND}_{\mathrm{Score}}+ \mathrm{PAB}_{\mathrm{Score}}+ \mathrm{KNO}_{\mathrm{Score}}}{6}$ |

**Table C.1:** Scoring procedure of the 31-items PPLQ version from stage 4 (i.e., PPLQ version 4)

***Note:*** The full wording of the corresponding item-labels can be retrieved from Table 1 in the manuscript.

**Table C.2:** Scoring procedure of the final 24-items PPLQ version from stage 5 (i.e., PPLQ version 5)

| **Domains scores** |  |
| --- | --- |
| Physical competence | **Coding:** 5 (= strongly agree) to 0 (= strongly disagree)  **Score calculation:**   1. $\mathrm{PCO}_{\mathrm{mean}}=\frac{\text{PCO\_ST1 + PCO \_EN1 + PCO\_ST2 +} \text{ PCO \_EN3}}{4}$ 2. $\mathrm{PCO}_{\mathrm{Score}}=\frac{\mathrm{PCO}_{\mathrm{mean}}}{5}\times100$ |
| Motivation | **Coding:** 5 (= strongly agree) to 0 (= strongly disagree)  **Score calculation:**   1. $\mathrm{MOT}_{\mathrm{mean}}=\frac{\text{(MOT\_IN1 × 2) + MOT \_ID1 + (MOT\_IN3 × 2) + MOT\_ID3}}{4}$ 2. $\mathrm{MOT}_{\mathrm{Score}}=\frac{\mathrm{MOT}_{\mathrm{mean}}}{7,5}\times100$ |
| Confidence  (self-efficacy) | **Coding:** 5 (= strongly agree) to 0 (= strongly disagree)  **Score calculation:**   1. $\mathrm{CON}_{\mathrm{mean}}=\frac{\text{CON\_IB2 + CON\_EB1 +} \text{CON\_EB2 + CON\_EB3}}{4}$ 2. $\mathrm{CON}_{\mathrm{Score}}=\frac{\mathrm{CON}_{\mathrm{mean}}}{5}\times100$ |
| Understanding | **Coding:** 5 (= strongly agree) to 0 (= strongly disagree)  **Score calculation:**   1. $\mathrm{UND}_{\mathrm{mean}}=\frac{\text{UND1 + UND2 + UND3}}{3}$ 2. $\mathrm{UND}_{\mathrm{Score}}=\frac{\mathrm{UND}_{\mathrm{mean}}}{5}\times100$ |
| Physical activity behavior | **Score calculation:**   1. $\mathrm{PAB}_{\mathrm{raw}}\text{= (PAB2a × PAB2b × 2) + (PAB3a × PAB3b) }$ 2. Truncation: Values exceeding 300 minutes are truncated to be equal to 300 minutes 3. $\mathrm{PAB}_{\mathrm{Score}}=\left( -\frac{4}{5625}\times\left( \mathrm{PAB}_{\mathrm{raw}} \right)^{2} \right)+\left( \frac{41}{75}\times\mathrm{PAB}_{\mathrm{raw}} \right)$ |
| Knowledge | **Coding:** all correct answers are coded with 1 (= 8 correct answers in total within the 4 items; incorrect answers are coded with 0; note that multiple answers are possible for the items KNO_KB1 and KNO_KB2, with each answer scored separately).  **Score calculation:**   1. $\mathrm{KNO}_{\mathrm{SUM}}\text{= }\text{KNO\_HM1 + KNO\_HM3 + KNO\_KB1 + KNO\_KB2 }$ 2. $\mathrm{KNO}_{\mathrm{Score}}=\frac{\mathrm{KNO}_{\mathrm{SUM}}}{8}\times100$ |
| **Total Physical Literacy Score** | **Score calculation:**  $\mathrm{PL}_{\mathrm{SCORE}}=\frac{\mathrm{PCO}_{\mathrm{Score}}\text{ + }\mathrm{MOT}_{\mathrm{Score}}\text{ + }\mathrm{CON}_{\mathrm{Score}}+ \mathrm{UND}_{\mathrm{Score}}+ \mathrm{PAB}_{\mathrm{Score}}+ \mathrm{KNO}_{\mathrm{Score}}}{6}$ |

***Note:*** The full wording of the corresponding item-labels can be retrieved from Table 1 in the manuscript.
