# Supplementary material for: Development of the Perceived Physical Literacy Questionnaire (PPLQ) for the adult population
Source: J Exerc Sci Fit. 2023 Oct 5;21(4):424–33. doi: 10.1016/j.jesf.2023.09.003 (PMC10661355; doi:10.1016/j.jesf.2023.09.003)
Supplement: Multimedia component 4 [file mmc4.docx]

| **Domains** | **χ^2^** | **df** | **χ^2^/df** | **CFI** | **CFI_Robust_** | **RMSEA (CI_90_)** | **RMSEA_Robust_ (CI_90_)** | **SRMR** |
| --- | --- | --- | --- | --- | --- | --- | --- | --- |
| Physical competence^a^ | 20.07 | 2 | 10,04 | 0.955 | 0.957 | 0.147 (0.098–0.202) | 0.165 (0.104–0.234) | 0.049 |
| Motivation^a^ | 4.94 | 2 | 2.47 | 0.993 | 0.993 | 0.059 (0.013–0.105) | 0.087 (0.000–0.188) | 0.017 |
| Confidence  (self-efficacy) | 1.47 | 2 | 0.74 | 1.000 | 1.000 | 0.000 (0.000–0.008) | 0.000 (0.00–0.102) | 0.011 |
| Knowledge^a^ | 28.00 | 20 | 1.40 | 0.988 | 0.945 | 0.031 (0.000–0.056) | 0.086 (0.000–0.148) | 0.071 |
| Understanding | n.a. | 0 | n.a. | n.a. | n.a. | n.a. | n.a. | n.a. |
| Physical activity behavior | n.a. | n.a. | n.a. | n.a. | n.a. | n.a. | n.a. | n.a. |

**Appendix D**

**Table D.1**

**Note:** ^a^The modification indices revealed residual error correlations between the items of the subfactors but were not freed in this one-factor model, which explains the global model misfit; χ^2^ = chi-squared; df = degrees of freedom; CFI = comparative fit index; RMSEA = root mean square error of approximation; CI_90_ = 90 percent confidence interval ; SRMR = standardized root mean square residual; When appropriate, MLR estimator was used instead of WLSMV; The domain Understanding (three indicators) showed insufficient fit for an essential tau-equivalent model. The congeneric model has 0 df, hence no model fit could be computed; The domain Physical activity behavior was not included in the analyses as it is represented in the model by only one indicator because of the formative nature of the physical activity construct (see Figure 1).
