# Supplementary material for: Development of the Perceived Physical Literacy Questionnaire (PPLQ) for the adult population
Source: J Exerc Sci Fit. 2023 Oct 5;21(4):424–33. doi: 10.1016/j.jesf.2023.09.003 (PMC10661355; doi:10.1016/j.jesf.2023.09.003)
Supplement: Multimedia component 5 [file mmc5.docx]

**Appendix E**

**Table E.1:** Correlations of PPLQ version 5 (i.e., 24-item version) and PAHCO

|  | **PPLQ** | | | | | | |
| --- | --- | --- | --- | --- | --- | --- | --- |
|  | **PCO** | **UND** | **MOT** | **CON** | **PAB** | **KNO** | **Overall PL** |
| **PAHCO: second-order-factors** |  |  |  |  |  |  |  |
| movement competence | 0.854 | 0.459 | 0.491 | 0.494 | 0.437 | 0.306 | 0.719 |
| control competence | 0.536 | 0.443 | 0.606 | 0.533 | 0.313 | 0.279 | 0.591 |
| self-regulation competence | 0.606 | 0.457 | 0.692 | 0.591 | 0.450 | 0.277 | 0.701 |
| **PAHCO: first-order-factors** |  |  |  |  |  |  |  |
| MED | 0.788 | 0.466 | 0.500 | 0.455 | 0.426 | 0.307 | 0.685 |
| MSD | 0.733 | 0.310 | 0.345 | 0.348 | 0.372 | 0.210 | 0.577 |
| MBD | 0.623 | 0.318 | 0.216 | 0.292 | 0.195 | 0.231 | 0.436 |
| Body Awareness | 0.451 | 0.386 | 0.430 | 0.348 | 0.228 | 0.175 | 0.436 |
| Control of Physical Load | 0.458 | 0.321 | 0.480 | 0.432 | 0.308 | 0.292 | 0.515 |
| Affect Regulation | 0.417 | 0.384 | 0.541 | 0.468 | 0.202 | 0.216 | 0.467 |
| Self-Efficacy | 0.699 | 0.313 | 0.499 | 0.501 | 0.467 | 0.224 | 0.652 |
| Self-Control | 0.500 | 0.383 | 0.639 | 0.573 | 0.429 | 0.221 | 0.632 |
| Emotional Attitudes | 0.363 | 0.423 | 0.644 | 0.432 | 0.284 | 0.222 | 0.504 |
| Cognitive Attitude | 0.187 | 0.335 | 0.353 | 0.292 | 0.078 | 0.291 | 0.316 |

***Note:*** n = 158 and p < .001 for all correlations; PCO: physical competence; UND: understanding; MOT: motivation; CON: confidence (self-efficacy); PAB: physical activity behavior; KNO: knowledge; PL: physical literacy; MED: Manageability of Endurance Demands; MSD: Manageability of Strength Demands.
